# Combined Treatment of Bronchial Epithelial Calu-3 Cells with Peptide Nucleic Acids Targeting miR-145-5p and miR-101-3p: Synergistic Enhancement of the Expression of the Cystic Fibrosis Transmembrane Conductance Regulator (*CFTR)* Gene

**DOI:** 10.3390/ijms23169348

**Published:** 2022-08-19

**Authors:** Chiara Papi, Jessica Gasparello, Matteo Zurlo, Alex Manicardi, Roberto Corradini, Giulio Cabrini, Roberto Gambari, Alessia Finotti

**Affiliations:** 1 Department of Life Sciences and Biotechnology, University of Ferrara, 44121 Ferrara, Italy; 2 Department of Chemistry, Life Sciences and Environmental Sustainability, University of Parma, 43124 Parma, Italy; 3 Research Center on Innovative Therapies for Cystic Fibrosis, University of Ferrara, 44121 Ferrara, Italy

**Keywords:** peptide nucleic acids, cystic fibrosis, microRNAs, miRNA targeting, miR-101-3p, miR-145-5p, CFTR

## Abstract

The Cystic Fibrosis Transmembrane Conductance Regulator (*CFTR*) gene encodes for a chloride channel defective in Cystic Fibrosis (CF). Accordingly, upregulation of its expression might be relevant for the development of therapeutic protocols for CF. MicroRNAs are deeply involved in the CFTR regulation and their targeting with miRNA inhibitors (including those based on Peptide Nucleic Acids, PNAs)is associated with CFTR upregulation. Targeting of miR-145-5p, miR-101-3p, and miR-335-5p with antisense PNAs was found to be associated with CFTR upregulation. The main objective of this study was to verify whether combined treatments with the most active PNAs are associated with increased *CFTR* gene expression. The data obtained demonstrate that synergism of upregulation of CFTR production can be obtained by combined treatments of Calu-3 cells with antisense PNAs targeting *CFTR*-regulating microRNAs. In particular, highly effective combinations were found with PNAs targeting miR-145-5p and miR-101-3p. Content of mRNAs was analyzed by RT-qPCR, the CFTR production by Western blotting. Combined treatment with antagomiRNAs might lead to maximized upregulation of CFTR and should be considered in the development of protocols for CFTR activation in pathological conditions in which *CFTR* gene expression is lacking, such as Cystic Fibrosis.

## 1. Introduction

MicroRNAs (miRNAs) are a class of short (19–25 nucleotides) noncoding RNAs that exhibit a very important role in post-transcriptional regulation of gene expression [1,2,3,4]. The control of gene expression by miRNAs is achieved by sequence-specific targeting of regulated mRNAs (most frequently within the 3′UTR), leading to a translational repression or mRNA degradation [1,2,3]. The miRNA/mRNA interaction occurs at the level of the RNA-induced Silencing Complex (RISC) [1,2,3]. The complex networks constituted by miRNAs and mRNA targets are the basis for the control of several biological functions, including cell growth, apoptosis, and differentiation [4,5,6]. A single microRNA might interact with several target mRNAs; conversely, a single 3′UTR mRNA sequence contains several functional miRNA binding sites [6]. Alterations of the miRNA/mRNA networks are associated with the onset and/or progression of several human pathologies [7,8,9,10]. In agreement, alteration of miRNA biological functions (either by miRNA inhibition or miRNA mimicking) is considered an interesting and innovative strategy with potential therapeutic implications [11,12,13,14,15]. This is not merely a theoretical possibility, since efforts aimed at translating laboratory investigations to clinical practice are in progress as demonstrated by the several ongoing clinical trials focusing on miRNA targeting in chronic hepatitis C, type 2 diabetes, cutaneous T-cell lymphoma (see, for instance, NCT01646489, NCT01200420, NCT02826525, and NCT02580552), and by an increasing number of reports and reviews related to this issue [16,17,18,19].

Several studies have been reported demonstrating that microRNAs are involved in Cystic Fibrosis (CF) [20,21,22,23,24,25,26,27,28], a genetic disease caused by alteration of production and/or biological activity of the gene coding the chloride channel CFTR (Cystic Fibrosis Transmembrane Conductance Regulator) [29,30]. Therefore, miRNA therapeutic approaches for CF are expected to have a great impact in the near future [27,28].

The role of microRNAs in CFTR regulation is well-established and has been recently explored by several research groups in different experimental model systems, including CF primary bronchial epithelial cells in vitro or bronchial brushings ex vivo [31,32,33,34,35,36,37,38,39,40,41,42,43,44,45,46,47,48,49,50]. For instance, Gillen et al. [31] identified at least 12 miRNAs (including miR-145 and miR-494) capable of repressing the expression of CFTR. In general, miRNA profiling strongly supported the concept that high expression of a set of miRNAs (directly interacting with the *CFTR* transcript) is associated with low expression of CFTR, as reported by Ramachandran et al. [35] in CF cells for miR-494 and miR-509. Oglesby et al. [36] confirmed and extended these observations in ex vivo analyses, demonstrating increased expression of miR-494, miR-223, and miR-145 in CF brushings of airway cells [36].

Our group has reported that miR-145-5p [38,39,45,47,49], miR-101-3p [46,49], and miR-335-5p [50] play an important role in regulating the expression of the *CFTR* gene. In particular, miR-145-5p has been demonstrated to regulate CFTR by several research groups, as demonstrated by Oglesby et al. [36], Fabbri et al. [38], Lutful Kabir et al. [41], and Dutta et al. [44]. A possible therapeutic application of these studies is that targeting these miRNAs (in our case miR-145-5p, miR-101-3p and miR-335-5p) with antagomiRNAs might be considered an experimental strategy to upregulate CFTR expression. Interestingly, the activity of different miRNAs on post-transcriptional *CFTR* gene regulation might be synergistic, as found by Megiorni et al. [32] for miR-101 and miR-494. In our recently published studies, we employed, as antagomiRNAs, Peptide Nucleic Acids (PNAs), DNA analogues of outstanding properties [51,52,53]. These molecules, despite a radical structural change with respect to DNA and RNA, are capable of sequence-specific and efficient hybridization with complementary DNA and RNA, forming Watson–Crick double helices [53,54]. Accordingly, PNAs and PNA-based analogues have been proposed as antisense molecules targeting mRNAs [55,56].

The main objective of the present study was to verify whether combined treatments with PNAs targeting miRNAs involved in CFTR regulation are associated with increased (possibly synergistic) *CFTR* gene expression. For this purpose, the effect of PNA mixtures on the content of miRNAs and mRNAs was analyzed by RT-qPCR, while the CFTR production by Western blotting.

The possibility of a synergistic action of antagomiRNAs targeting different microRNAs might retain important impact in the development of protocols of interest in translating laboratory findings into therapeutic applications.

## 2. Results

### 2.1. The MicroRNAs miR-145-5p, miR-101-3p, and miR-335-5p Regulate Different Target mRNAs

**Figure 1:**
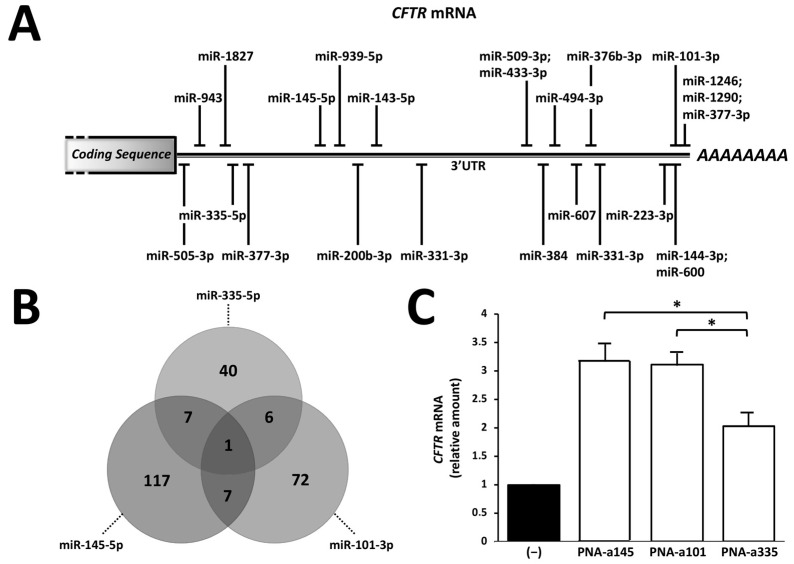
Characterization of the biological activity of miR-145-5p, miR-101-3p, and miR-335-5p on *CFTR* gene expression. (**A**) microRNA binding sites present within the 3′UTR of *CFTR* mRNA; (**B**) Venn-diagram indicating the number of mRNA targets of miR-145-5p, miR-101-3p, and miR-335-5p: only *CFTR* mRNA is a molecular target of all the three microRNAs. Analysis of mRNAs targeted by miRNAs was conducted using TargetScan (release 8.0) and data already in the literature. Afterwards, a Venn-diagram was performed using a Draw Venn Diagram tool (http://bioinformatics.psb.ugent.be/webtools/Venn/, accessed on 9 May 2022); (**C**) upregulation of *CFTR* mRNA following treatment of Calu-3 cells with PNAs against miR-145-5p (PNA-a145), miR-101-3p (PNA-a101) and miR-335-5p (PNA-a335). *n* = 3 independent RT-qPCR experiments (* indicates differences with *p* < 0.05).

Figure 1A summarizes the binding sites for microRNAs present within the 3′UTR of the *CFTR* mRNA. This scheme is based on several recently published reports [31,32,33,34,35,36,37,38,39,40,41,42,43,44,45,46,47,48,49,50,57]. The list of these miRNA binding sites, the location of their most probable binding site(s) within the 3′UTR *CFTR* mRNA sequence, and the studies originating this information are presented in Table S1. As clearly evident, several functional sites are present that can be the object of novel approaches in miRNA-therapeutics, including combined treatments.

Most of these sites have been shown to regulate *CFTR* mRNA stability and production of CFTR protein.

Our group has demonstrated that, among these microRNAs, miR-145-5p, miR-101-3p, and miR-335-5p efficiently and selectively bind the 3′UTR sequence of *CFTR* mRNA. Inhibition of their interaction with *CFTR* mRNA leads to upregulation of *CFTR* mRNA content and CFTR protein accumulation. On the contrary, PNA-based miRNA inhibitors targeting other microRNAs (such as miR-433-3p and miR-509-3p) were found to be less efficient in our experimental Calu-3 cell system. For this reason, in this Short Report, we focused our attention on PNAs inhibiting miR-145-5p, miR-101-3p, and miR-335-5p. Figure 1B shows a computer-aided analysis demonstrating that miR-145-5p, miR-101-3p, and miR-335-5p are able to target different sets of mRNAs. Interestingly, *CFTR* mRNA was found to be targeted, unlike the other mRNAs, by these three microRNAs. This analysis was performed using the list of the mRNAs targeted by miR-145-5p, miR-101-3p, and miR-335-5p, presented in Table S2, which includes the relative publications.

### 2.2. CFTR Expression Depends on MicroRNAs miR-145-5p, miR-101-3p, and miR-335-5p

When bronchial epithelial Calu-3 cells were cultured in the presence of PNAs targeting miR-145-5p, miR-101-3p, and miR-335-5p (PNA-a145, PNA-a101, and PNA-a335, respectively), an upregulation of *CFTR* mRNA was observed when RT-qPCR was performed, as outlined in the representative example shown in Figure 1C. In order to obtain efficient transfection levels, in our studies, PNAs were functionalized with a R8-polyarginine peptide. This strategy allows the uptake of the majority of the PNA molecules within few hours, reaching a maximum level (near 100%) after 72 h, as firstly published by Brognara et al. [58] and then confirmed by several studies of our group [38,39,46,50]. The issue of PNA delivery has been fully addressed in a recent review paper by Volpi et al. [59].

These data obtained are in good agreement with published reports by Oglesby et al. [36], Fabbri et al. [38], Gambari et al. [39], Lutful Kabir et al. [41], and Dutta et al. [44] on antagomiRNAs against miR-145-5p, by Megiorni et al. [32], Hassan et al. [33], and Fabbri et al. [46] on antagomiRNAs against miR-101-3p, and by Tamanini et al. on antagomiRNAs against miR-335-5p [50]. The selectivity of the effects of PNA-a145, PNA-a101, and PNA-a335 on the expression of miR-145-5p, miR-101-3p, and miR-335-5p has been already discussed elsewhere by Fabbri et al. [38], Fabbri et al. [46], and Tamanini et al. [50]. On the basis of the data discussed in Figure 1, combined treatments based on PNA-a145 and PNA-a101 were performed on Calu-3 cells.

### 2.3. Combined Treatment Based on PNAs against miR-145-5p and miR-101-3p: Effects on Cell Growth of Calu-3 Cells and on CFTR mRNA Accumulation

**Figure 2:**
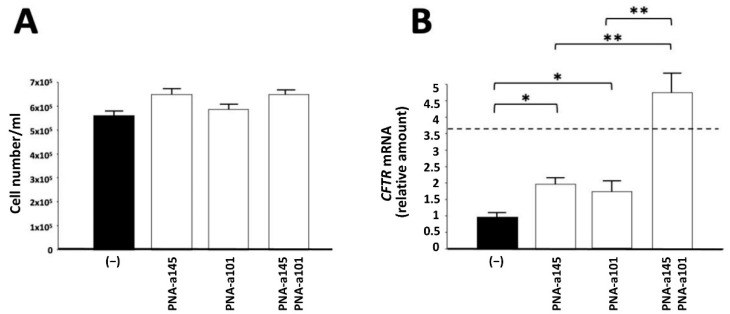
Effects on cell proliferation and *CFTR* mRNA accumulation of combined treatment of Calu-3 cells with PNAs against miR-145-5p and miR-101-3p. Calu-3 cells were treated for three days with PNA-a145 and PNA-a101, administered either singularly, or in combination. After these treatments, cell number/mL was determined, and *CFTR* mRNA content was analyzed by RT-qPCR. (**A**) effects on cell growth; (**B**) effects on *CFTR* mRNA. The results of (**B**) represent the fold change of the *CFTR* with respect to control untreated Calu-3 cells (*n* = 3 independent RT-qPCR experiments; * *p* < 0.05, ** *p* < 0.01). The dotted line represents the sum of the values obtained with single administrations of PNA-a145 and PNA-a101.

In the experiment shown in Figure 2, the effects of combined treatment with PNA-a145 and PNA-a101 are reported, which are the PNAs that, when administered singularly, have been shown to increase the *CFTR* levels more efficiently.

**Figure 3:**
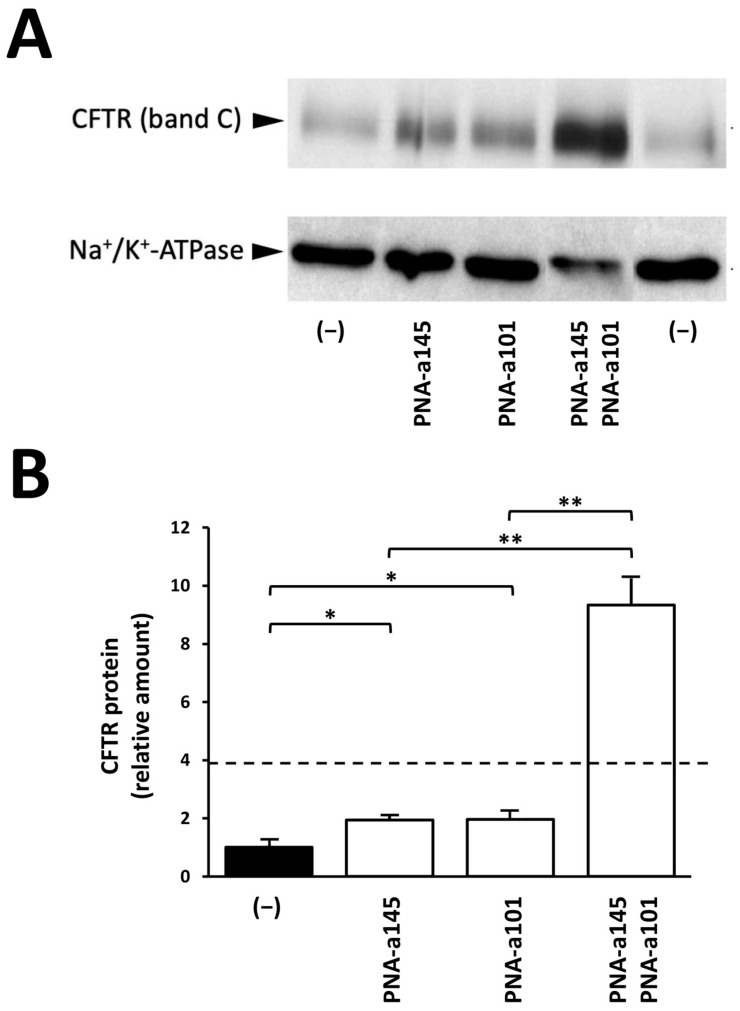
Effects of combined treatment of Calu-3 cells with PNAs against miR-145-5p and miR-101-3p on CFTR protein accumulation. Calu-3 cells were treated for three days with PNA-a145 and PNA-a101, administered either singularly, or in combination. After these treatments, proteins were isolated and Western blotting was performed. (**A**) representative results of Western blotting performed using monoclonal antibodies recognizing CFTR or Na^+^/K^+^-ATPase, as indicated; (**B**) summary of the experiments performed. Results represent the mean fold changes with respect to the control untreated cells of the CFTR/Na^+^/K^+^-ATPase ratios (*n* = 3 independent quantifications of the same blot; * *p* < 0.05, ** *p* < 0.01). The uncut version of the Western blotting gels of (**A**) and the relative Ponceau staining are included in Supplementary materials (Figure S1). The dotted line represents the sum of the values obtained with single administrations of PNA-a145 and PNA-a101.

No major inhibitory effects on cell proliferation of Calu-3 cells were observed. The cell number/mL was determined after three days of cell culture. Each cell culture was started using a Calu-3 cell concentration of 200,000 cells/mL (day 0) (Figure 2A). On the contrary, when *CFTR* mRNA was quantified by RT-qPCR, an increase was detectable in the combined treatments. The fact that treatment with PNA-based antagomiRNAs leads to alteration of *CFTR* mRNA accumulation is expected, as elsewhere discussed [38,39,46,50]. Therefore, a more extensive analysis was performed by Western blotting, as shown in Figure 3.

### 2.4. Combined Treatments Based on PNAs against miR-145-5p and miR-101-3p: Synergistic Effects on CFTR Protein Accumulation

Calu-3 cells were cultured in the presence of PNAs against miR-145-5p and miR-101-3p, administered singularly or in combination. After 72 h of treatment, proteins were isolated from the cells, and Western blotting was performed using two antibodies, one against CFTR (the mouse monoclonal antibody, clone 596, against NBD2 domain of CFTR) and the other against the house-keeping internal control Na^+^/K^+^-ATPase. A representative Western blotting is shown in Figure 3A, while the summary of multiple determinations is shown in Figure 3B. The uncut version of the Western blotting autoradiogram generating Figure 3A has been included in Supplementary materials (Figure S1).

The data obtained show that the combined treatment based on co-administration of PNA-a145 and PNA-a101 exhibits high efficiency in inducing the increased expression of CFTR. The obtained levels of CFTR content in combined PNA-a145/PNA-a101 treatments were higher than those predicted considering the sum of the respective values of CFTR content obtained using singularly administered PNA-a145 and PNA-a101. For example, the fold increase of CFTR was 1.94 and 1.96 in Calu-3 cells treated with PNA-a145 and PNA-a101, respectively. The increase of CFTR in PNA-a145 plus PNA-a101 combined treatment was 9.33, a value that is 2.4-fold higher than the addition of the values obtained using single treatments based on PNA-a145 and PNA-a101 (i.e., 3.9). According to the approach described by Chou and Talalay [60,61], these results indicate synergistic (rather than additional) effects.

## 3. Discussion

Since the demonstration that microRNAs are deeply involved in the regulation of the *CFTR* gene [31,32,33,34,35,36,37,38], a great attention has been dedicated to possible alteration of *CFTR* gene expression by targeting those miRNAs causing downregulation of this gene. For instance, PNA-mediated inhibition of the CFTR-regulators miR-145-5p, miR-101-3p and miR-335-5p leads to CFTR increase in the Calu-3 model system [28,39,46,50]. The upregulation of CFTR using antagomiRNA strategies has been also reported in a limited number of studies, such as those published by Oglesby et al. [36] and by Amato et al. [37]. Oglesby et al. found that transfection to CFBE41o− cells of molecules designed to knock down miR-145, miR-223, and miR-494 alone and in combination, leads to a reciprocal increase in *CFTR* mRNA expression. When all three miRNAs are knocked down in combination, CFTR expression is increased, but not in a synergistic fashion [36]. Amato et al. exploited a PNA targeting miR-509-3p and demonstrated that it causes an increase of luciferase activity in A549 cells co-transfected with a pLUC-CFTR-3′UTR-wt vector and miR-509-3p [37].

The possibility to upregulate CFTR expression is of great relevance in the possible personalized treatment of Cystic Fibrosis, a genetic disease that is characterized by a deep alteration of CFTR. In this context, the possibility of PNA-dependent modulation of miRNAs involved in the regulation of CFTR expression is of great impact in the case this approach will be demonstrated to be efficient in further increasing the activity of drugs already employed for personalized therapy of CF, such as, for instance, read-through molecules [62,63,64] and CFTR correctors [65,66,67]. This combined treatment might lead to unmet levels of functional CFTR production in CF cells, through the combination of two complementary therapeutic relevant activities, i.e., the miRNA-dependent upregulation of CFTR production and the use of personalized drugs.

The main result of our study is the demonstration that combined treatments of Calu-3 cells with molecules targeting different CFTR-regulating miRNAs might lead to a synergistic action on CFTR upregulation. This was found with combined treatments based on PNA-a145 and PNA-a101. Possible synergistic effects of miRNAs on *CFTR* have also been proposed in the past. For instance, synergistic post-transcriptional regulation of the CFTR by miR-101 and miR-494 specific binding was firstly reported by Megiorni et al. [32]. In their study, HEK293 cells were co-transfected either with a reporter construct containing 741 base pairs of the human *CFTR* 3′UTR in the presence of a synthetic microRNA mimic (miR-101 or miR-494). While both miR-101 and miR-494 significantly suppressed luciferase expression, when they were co-overexpressed, a synergistic effect between miRNAs was observed.

The knowledge that miRNAs might inhibit CFTR is the basis of a rational approach to upregulate CFTR by means of using anti-miRNA molecules (such as those based on PNAs used by our research group) or interfering with the miRNA/*CFTR* interactions using *CFTR*-specific target site blockers (TSBs) able to “mask” the miRNA binding sites present within the 3′UTR of the *CFTR* mRNA (see Figure 1A) [47,48,68]. In particular, the first report on the use of PNAs as target site blockers in cystic fibrosis was published by Zarrilli et al., who tested the ability of PNAs fully complementary to the *CFTR* 3′UTR sequence recognized by miR-509-3p, in order to rescue the CFTR expression in the A549 cellular model co-transfected with a pLuc-CFTR-3′UTR vector [68]. This “masking” approach was then fully validated by Sultan et al. [47] and by De Santi et al. [48].

Our study is the proof-of-principle that combined treatments (using different miRNA inhibitors or TBS) might be necessary for obtaining very high CFTR upregulation. This is a pre-requisite to develop efficient biomedicines for CFTR therapy. With respect to clinical relevance of the approach presented in this study, it is expected that decreased availability of miRNAs (as in the anti-miR approach) is associated with an accumulation and translation of target mRNAs (in our case *CFTR* mRNA).

Experimental trials based on miRNA targeting are ongoing. A first example is constituted by the Phase 2 trial, using miravirsen (Santaris Pharma, Copenhagen, Denmark, NCT01200420) and the Phase I trial, using RG-101 (Regulus Therapeutics, San Diego, CA, USA, NCT00980161), both based on targeting miR-122 for therapy of Hepatitis C Virus infection. Another example is a study of RGLS4326 in patients with autosomal dominant Polycystic Kidney Disease (NCT04536688, by Regulus Therapeutics, San Diego, CA, USA), targeting miR-17. In this context, PNAs represent molecules with strong anti-miRNA activity, although there are few clinical trials, in this case. Reviews on clinical trials based on miRNA therapeutics have been recently published [15,69,70].

While the results presented in this Brief Report are in our opinion promising, for our study, further improvements can be envisaged, namely: (a) since many other miRNA binding sites are present within the 3′UTR *CFTR* mRNA sequence (see Figure 1A), combinatorial studies can be performed in order to identify the best mixture for CFTR upregulation; (b) triple- or multi-combination might be also considered; (c) PNAs targeting multiple miRNAs might also display improved effects on CFTR; and (d) co-treatments with CFTR correctors/potentiators and/or with read-through molecules can be explored.

Regarding point (a) and in consideration of our recent interest in regulation of *CFTR* gene expression by miR-335-5p [50], the possible synergism between PNA-a145 and PNA-a335 was also considered (Table S3). We found that the combined treatment with PNA-a145 and PNA-a335 leads to a more efficient CFTR upregulation (4.95 fold) than the singular treatments (1.94 fold and fold 1.57, respectively). The effect on CFTR increase is less efficient than that obtained with the combination between PNA-a145 and PNA-a101. No increase was found with combination PNA-a101 and PNA-a335, compared to that derived from the single treatments.

In conclusion, despite the fact that the combinations used were limited, the present study represents a proof of principle that co-targeting of multiple miRNAs might lead to high induction of CFTR.

This might open a novel avenue in the personalized treatments of CF. In fact, in addition to personalized treatments considering the *CFTR* mutation of the CF patients under treatment, different dosage of the anti-miRNA components can be tailored depending on the pattern of CFTR-regulating miRNAs upregulated in the CF patients subject to further optimization of the intervention based on these precision medicines.

Several issues remain to be studied in depth, i.e., how much synergism can be obtained using different doses of PNA-a145 and PNA-a101. Moreover, a key issue is how many folds of dose-reduction for each drug (PNA-a145 and PNA-a101) can be proposed as a result of synergism. This is a key factor predicting for toxicity reduction to be considered in the design of protocols for clinical trials. The studied PNAs have been demonstrated, under the experimental conditions used in this Brief Report, to be unable to induce increase of the proportion of the dead cell population in treated Calu-3 cells. With respect to this point, these PNAs have been previously demonstrated by FACS analyses to be unable to induce apoptosis and increase of the proportion of the dead cell population in treated Calu-3 cells [38,46]. Apoptosis assays have been performed using the Muse Cell Analyzer instrument (Millipore Corporation, Billerica, MA, USA). The Muse Annexin V and Dead Cell Kit and the Muse Caspase-3/7 Kit were employed [15,17]. These assays differentiate viable non-apoptotic cells from early apoptotic, late apoptotic, and dead cells. Even more importantly, a recently published NGS study demonstrated that PNA-a145 and PNA-a101 do not alter the miRNome of treated Calu-3 cells [49], supporting the concept that they have no (or limited) effect on the overall transcriptional machinery of treated cells. Altogether, these studies suggest that the PNAs used here are not toxic to Calu-3 cells, fully in agreement with the data presented in Figure 2A, demonstrating that PNA-a145 and PNA-a101 do not alter the proliferation rate of Calu-3 cells when used either singularly or in combination. A final unanswered question is what are the best combinations considering PNAs targeting all the miRNAs regulating CFTR expression (see Table S2) through a binding to the 3′UTR (see Figure 1A). Despite the fact that this study is still ongoing, the present Brief Report sustains the concept that combined treatments targeting multiple miRNAs might be considered to maximize CFTR pharmacological upregulation.

## 4. Materials and Methods

### 4.1. Synthesis and Characterization of PNAs

Synthesis and characterizations of anti-miRNA PNAs were previously reported [38,39,46,47,50]. All PNA sequences have an octaarginine tail (R8), for the delivery into the cells without other carriers [12,59]. After synthesis and cleavage from the solid support, PNA purification was performed by HPLC using a Phenomenex Jupiter RPC18, (250 × 4.6 mm, 1.7 μm) column as described elsewhere [38]. After purification, the PNAs’ identity and purity were confirmed by UPLC/ESI-MS (Waters Acquity ultra performance LC HO6UPS-823M, with Waters SQ detector equipped with Waters UPLC BEH C18, 50 × 2.1 mm, 1.7 μm) at 35 °C. The concentration of the PNA was determined using UV-absorbance at 260 nm assuming an additive contribution of nucleobases. The sequence of the PNAs employed in this study is reported in Table 1. The PNAs were designed according to our standardized protocols, using the following criteria: (a) length of 18 bp, suitable for efficient synthesis also on large scale; (b) lack of self-complementarity both in antiparallel and parallel orientation; (c) minimal length of complementary sequences in mRNA, as evaluated by BLAST search; (d) when possible, targeting of the “seed region” which is an essential element for miRNA function [12,39]. A carrier octaargine (R8) peptide was conjugated at N-terminus of the PNA chain since it induces an efficiency in the delivery which approaches 100% (i.e., uptake in 100% of the target cell population), as elsewhere published [12,38,47,50,58]; this conjugation is easily realized during PNA solid-phase synthesis using the same reagents and solvents. We have previously reported the higher efficiency of R8-PNA conjugates (R8-PNAs) in inhibiting target miRNAs when this activity is compared to that of conventional commercially available antagomiRNAs [38,39]. The possible formation of heterodimers between the different PNA probes has been further evaluated using mfold and Multiple Primer Analyzer webtools. In all cases, no significant interactions were observed.

### 4.2. Cell Lines and Culture Conditions

The bronchial epithelial Calu-3 cells [38,71] were cultured in a humidified atmosphere of 5% CO_2_/air in DMEM/F12 medium (Gibco, Grand Island, NY, USA) supplemented with 10% fetal bovine serum (Biowest, Nauillè, France), 100 units/mL penicillin, 100 μg/mL streptomycin (Lonza, Verviers, Belgium), and 1% NEEA (100X) (Non-Essential Amino Acids Solution; Gibco, Grand Island, NY, USA). To determine the effect on proliferation, cell growth was monitored by determining the cell number/mL using a Z2 Coulter Counter (Coulter Electronics, Hialeah, FL, USA).

### 4.3. Cell Treatments with PNAs

Calu-3 cell lines were seeded in a 12-well plate with a concentration of 200,000 cells/mL. The day after the seeding, cells were treated with PNA-a145 2 μM, PNA-a101, and PNA-a335 4 μM, singularly or in combination. Cellular uptake with these R8-PNAs did not require additional transfection agents. After 72 h cells were collected, washed with cold sterile PBS, and total cell extracts and RNA were prepared.

### 4.4. RNA Extraction

Cultured cells were trypsinized and collected by centrifugation at 1500 rpm for 10 min at 4 °C, washed with PBS, lysed with Tri-Reagent (Sigma Aldrich, St. Louis, MI, USA), according to manufacturer’s instructions. The isolated RNA was washed once with cold 75% ethanol, dried, and dissolved in nuclease free pure water before use [38,39,46,50].

### 4.5. Analysis of CFTR Expression: RT-qPCR

Gene expression analysis was performed by RT-qPCR using 300 ng of total RNA, extracted, and reverse transcribed using the Taq-Man Reverse Transcription PCR Kit and random hexamers (Applied Biosystems, Waltham, MA, USA) as RT reaction primers. Quantitative real-time PCR (RT-qPCR) assays were carried out using gene-specific double fluorescently labeled probes. Primers and probes used to assay *CFTR* (Assay ID: Hs00357011_m1) gene expression were purchased from Applied Biosystems. The relative expression was calculated using the comparative cycle threshold method and, as reference genes, the human RPL13A (Assay ID: Hs03043885_g1).

### 4.6. Analysis of CFTR Expression: Western Blotting

CFTR expression was measured using Western blotting analyses. Cell pellets were lysed in RIPA buffer (Thermo Fisher Scientific, Waltham, MA, USA) and sonicated for 30 s three times on ice at 50% amplitude using the Vibra-Cell VC130 Ultrasonic Processor (Sonics). Lysates were cleared by centrifugation at 14,000× *g* for 30 min at 4 °C. Protein concentration was determined by BCA Protein Assay Kit (Thermo Fisher Scientific, Waltham, MA, USA) according to the manufacturer’s protocol. For CFTR analysis, 40 μg of total protein extracts were heated in Blue Loading Buffer by adding 50 nM of dithiothreithol (DTT) (Cell Signaling Technology, Danvers, MA, USA) at 37 °C for 10 min and loaded onto a 7% SDS-polyacrylamide gel. Then, the gel proteins were transferred to nitrocellulose membrane (Thermo Fischer Scientific, Waltham, MA, USA) by using Trans-Blot Turbo (Bio-Rad Laboratories, Hercules, CA, USA) and gross loading errors were excluded by staining of the gels with Ponceau S solution, as depicted in Figure S1 (bottom panel). Membranes was processed for Western blotting by using mouse monoclonal anti-body, clone 596, against NBD2 domain of CFTR (University of North Carolina, Cystic Fibrosis Center, Chapel Hill, NC, USA) at a dilution of 1:2,500 by overnight incubation at 4 °C. After washes, membranes were incubated with horseradish peroxidase-coupled anti-mouse immunoglobulin (R&D System, Minneapolis, MN, USA) at room temperature for 1 h and, after washes, the signal was developed by enhanced chemiluminescence (LumiGlo Reagent and Peroxide, Cell Signaling Technology, Danvers, MA, USA). The acquisition of the membrane images and the densitometric values of the bands were obtained using the ChemiDoc Imaging Systems (Bio-Rad Laboratories, Hercules, CA, USA). After membrane stripping, Na^+^/K^+^-ATPase α1 monoclonal antibody (sc-514614, Santa Cruz Biotechnology, Dallas, TX, USA) was used to compare the CFTR content with that of an internal control (Na^+^/K^+^-ATPase), a widely accepted strategy to assess changes of gene expression in treated cells by Western blotting [72]. The relative amount of CFTR protein was calculated after normalization of the densitometric values of CFTR bands with the densitometric values obtained using the anti-Na^+^/K^+^-ATPase antibody. In particular, for the sample loading normalization, the CFTR protein signal in each lane is divided (normalized) by the signal value for the Na^+^/K^+^-ATPase in that lane, and then relative levels of CFTR protein were compared across the blot using untreated cells (−) as a reference sample [72]. Na^+^/K^+^-ATPase was selected as an internal control for two main reasons: (a) its molecular weight is in the range of CFTR (100 kDa for Na^+^/K^+^-ATPase α1; 170–180 kDa for CFTR band C), allowing the use of the same Western blotting filter; (b) its protein extraction, purification, and pre-loading denaturation protocol are very similar to that employed for CFTR. Of course, as suggested elsewhere [72], our conclusion might be further supported by the use of additional internal protein controls (GAPDH, β-actin and others).

### 4.7. Statistical Analysis

Results are expressed as mean ± standard error of the mean (SEM). Comparisons between groups were made by using one-way ANOVA (* *p* < 0.05, ** *p* < 0.01, *** *p* < 0.001 and **** *p* < 0.0001).

## Figures and Tables

**Table 1:** PNA sequence employed in Calu-3 cells treatment.

PNA	Sequence	MicroRNA Target
PNA-a101	H-R8-AGTTATCACAGTACTGTA-Gly-NH2	miR-101-3p
PNA-a335	H-R8-TTTCGTTATTGCTCTTGA-Gly-NH2	miR-335-5p
PNA-a145	H-R8-AGGGATTCCTGGGAAAAC-Gly-NH2	miR-145-5p

## Data Availability

The datasets and materials generated and/or analyzed during the present study are available from the corresponding author upon reasonable request.

## Supplementary Materials

The supporting information can be downloaded at: https://www.mdpi.com/article/10.3390/ijms23169348/s1.
