# Supplementary material for: Combined Treatment of Bronchial Epithelial Calu-3 Cells with Peptide Nucleic Acids Targeting miR-145-5p and miR-101-3p: Synergistic Enhancement of the Expression of the Cystic Fibrosis Transmembrane Conductance Regulator (CFTR) Gene
Source: Int J Mol Sci. 2022 Aug 19;23(16):9348. doi: 10.3390/ijms23169348 (PMC9409490; doi:10.3390/ijms23169348)
Supplement: Supplementary file 1 [file ijms-23-09348-s001.zip › ijms-1830196-supplementary.pdf]

Combined treatment of bronchial epithelial Calu-3 cells with Peptide Nucleic Acids Targeting miR-145-5p and miR-101-3p: Synergistic Enhancement of the Expression of the Cystic Fibrosis Transmembrane Conductance Regulator (*CFTR*) Gene

Chiara Papi[1], Jessica Gasparello[1], Matteo Zurlo[1], Alex Manicardi[2], Roberto Corradini[2], Giulio Cabrini[1,3], Roberto Gambari[1,3,*] and Alessia Finotti[1,3]

[1] Department of Life Sciences and Biotechnology, University of Ferrara, Italy
[2] Department of Chemistry, Life Sciences and Environmental Sustainability, University of Parma, Italy;
[3] Research Center on Innovative Therapies for Cystic Fibrosis, University of Ferrara, Italy

* Correspondence: gam@unife.it; Tel.: +39-0532-974443 (R.G.)

SUPPLEMENTARY MATERIALS

FIGURE S1

Uncut version of the Western blotting data

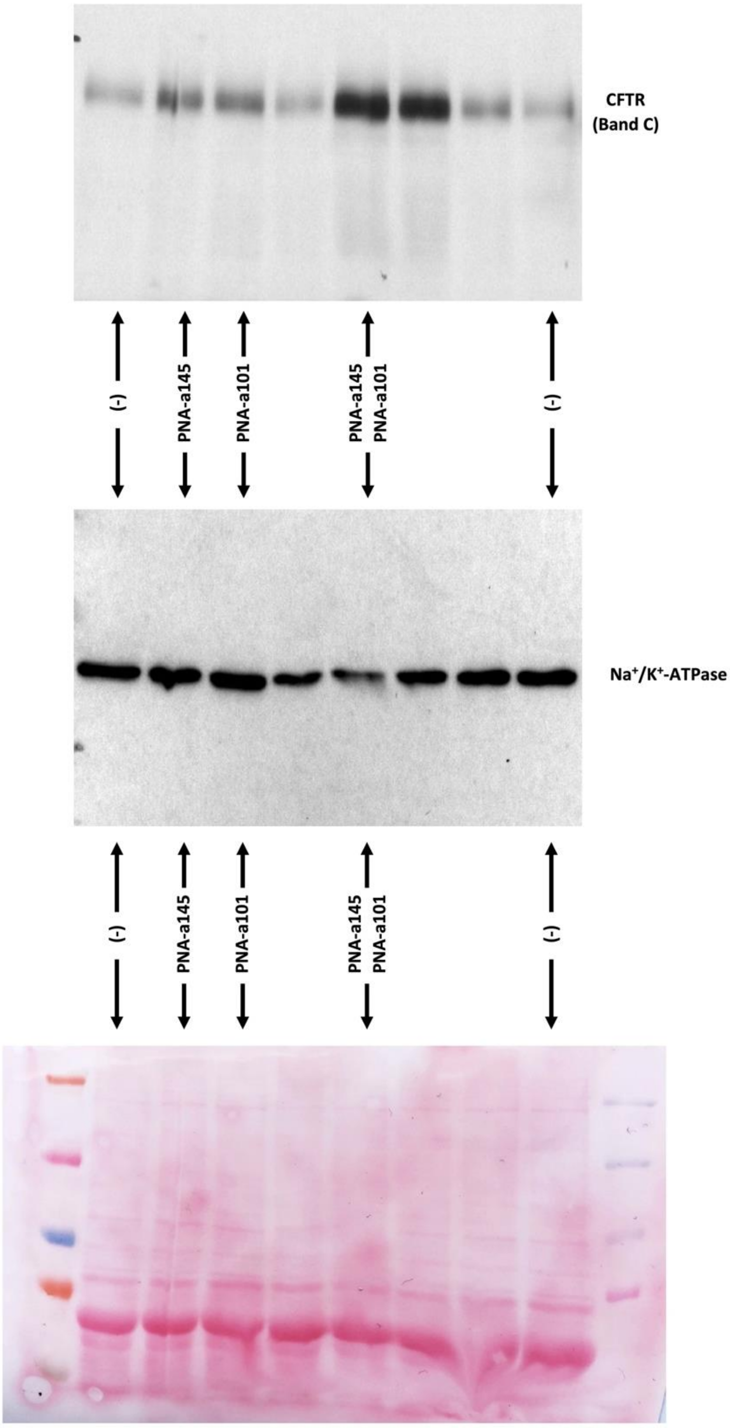

These data have been used for Figure 3A, upper part of the panel (up) and Figure 3A, lower part of the panel (down).

TABLE S1

List of miRNAs targeting CFTR and location of their binding sites within the CFTR 3′UTR sequence

miRNA	Binding sites	Reference
miR-505-3p	15 - 21	Viart et al., 2015
miR-943	56 - 62	Viart et al., 2015
miR-1827	97 - 104	Gillen et al., 2011
miR-335-5p	170 - 183	Tavazoie et al., 2008
miR-377-3p	212 – 219<br>1549 - 1556	Viart et al., 2015
miR-145-5p	427 - 434	Gilllen et al., 2011<br>Oglesby et al., 2013<br>Viart et al., 2015<br>Fabbri et al., 2017<br>Lutful et al., 2017
miR-939-5p	477 - 483	Gilllen et al., 2011
miR-200b-3p	545 - 551	Bartoszewska et al., 2017
miR-143-5p	573 - 580	De Santi et al., 2018
miR-331-3p	740 – 746<br>1304 - 1310	Gilllen et al., 2011
miR-433-3p	1060 - 1067	Amato et al., 2013
miR-509-3p	1056 - 1063	Amato et al., 2013<br>Ramachandran et al., 2013
miR-384	1118 - 1125	Gilllen et al., 2011
miR-494-3p	1140 - 1147	Gilllen et al., 2011<br>Viart et al., 2015<br>Megiorni et al., 2011<br>Oglesby et al., 2013<br>Ramachandran et al., 2013

miR-607	1216 - 1223	Gilllen et al., 2011
miR-376b-3p	1250 - 1257	Gilllen et al., 2011
miR-223-3p	1475 - 1481	Oglesby et al., 2013
miR-101-3p	1508 - 1515	Megiorni et al., 2011<br>Hassan et al., 2012<br>Viart et al., 2015
miR-144-3p	1508 - 1514	Hassan et al., 2012
miR-600	1510 - 1517	Gilllen et al., 2011<br>Viart et al., 2015
miR-1290	1535 - 1542	Gillen et al., 2011
miR-1246	1537 - 1544	Gilllen et al., 2011<br>Lukosevicius et al., 2022

References

Amato, F.; Seia, M.; Giordano, S.; Elce, A.; Zarrilli, F.; Castaldo, G.; Tomaiuolo, R. Gene mutation in microRNA target sites of CFTR gene: a novel pathogenetic mechanism in cystic fibrosis? *PLoS One* **2013**, 8(3):e60448; Doi: 10.1371/journal.pone.0060448.

Bartoszewska, S.; Kamysz, W.; Jakiela, B.; Sanak, M.; Kroliczewski, J.; Bebok, Z.; et al. miR-200b downregulates CFTR during hypoxia in human lung epithelial cells. *Cell. Mol. Biol. Lett.* **2017**, 22:23; Doi: 10.1186/s11658-017-0054-0.

De Santi, C.; Gadi, S.; Swiatecka-Urban, A.; Greene, C. Identification of a novel functional miR-143-5p recognition element in the Cystic Fibrosis Transmembrane Conductance Regulator 3'UTR. *AIMS Genet.* **2018**, 23;5(1):53-62; Doi: 10.3934/genet.2018.1.53.

Fabbri, E.; Tamanini, A.; Jakova, T.; Gasparello, J.; Manicardi, A.; Corradini, R.; et al. A peptide nucleic acid against MicroRNA miR-145- 5p enhances the expression of the cystic fibrosis transmembrane conductance regulator (CFTR) in Calu-3 cells. *Molecules* **2017**, 23:E71; Doi: 10.3390/molecules23010071.

Gillen, A.E.; Gosalia, N.; Leir, S,H.; Harris, A. Microrna regulation of expression of the cystic fibrosis transmembrane conductance regulator gene. *Biochem. J.* **2011**, 438, 25–32; Doi:10.1042/BJ20110672.

Hassan, F.; Nuovo, G.J.; Crawford, M.; Boyaka, P.N.; Kirkby, S.; Nana-Sinkam, S.P.; et al. MiR-101 and miR-144 regulate the expression of the CFTR decreases interleukin-8 production. *Eur. Respir. J.* **2012**, 46, 1350–1360; Doi: 10.1183/ 09031936.00163414.

Lukosevicius, R.; Juzenas, S.; Salteniene, V.; Kulokiene, U.; Arstikyte, J.; Hemmrich-Stanisak, G.; et al. miRNome Profiling and Functional Analysis Reveal Involvement of hsa-miR-1246 in Colon Adenoma-

Carcinoma Transition by Targeting AXIN2 and CFTR. *Int. J. Mol. Sci.* **2022**, 14;23(4):2107; Doi: 10.3390/ijms23042107.

Lutful Kabir, F.; Ambalavanan, N.; Liu, G.; Li, P.; Solomon, G.M.; Lal, C.V.; et al. MicroRNA-145 antagonism reverses TGF-beta inhibition of F508del CFTR correction in airway epithelia. *Am. J. Respir. Crit. Care Med.* **2017**, 197, 632–643; Doi: 10.1164/rccm.201704-0732OC.

Megiorni, F.; Cialfi, S.; Dominici, C.; Quattrucci, S.; Pizzuti, A. Synergistic post-transcriptional regulation of the Cystic Fibrosis Transmembrane conductance Regulator (CFTR) by miR-101 and miR- 494 specific binding. *PLoS One* **2011**, 6:e26601; Doi: 10.1371/journal.pone.002 6601.

Oglesby, I.K.; Chotirmall, S.H.; McElvaney, N.G.; Greene, C.M. Regulation of cystic fibrosis transmembrane conductance regulator by MicroRNA-145, -223, and -494 is altered in DeltaF508 cystic fibrosis airway epithelium. *J. Immunol* **2013**, 190, 3354–3362; Doi: 10.4049/jimmunol.120 2960.

Ramachandran, S.; Karp, P.H.; Osterhaus, S.R.; Jiang, P.; Wohlford-Lenane, C.; Lennox, K.A.; et al. Post-Transcriptional regulation of cystic fibrosis transmembrane conductance regulator expression and function by MicroRNAs. *Am. J. Respir. Cell Mol. Biol*. **2013**, 49, 544–551; Doi: 10.1165/rcmb.2012-0430OC.

Tavazoie, S.F.; Alarcón, C.; Oskarsson, T.; Padua, D.; Wang, Q.; Bos, P.D.; et al. Endogenous human microRNAs that suppress breast cancer metastasis. *Nature* **2008**, 10;451(7175):147-52; Doi: 10.1038/nature06487.

Viart, V.; Bergougnoux, A.; Bonini, J.; Varilh, J.; Chiron, R., Tabary, O.; et al. Transcription factors and miRNAs that regulate fetal to adult CFTR expression change are new targets for cystic fibrosis. *Eur. Respir. J.* **2015**, 45, 116–128; Doi: 10.1183/09031936.00113214.

TABLE S2

List of the mRNAs targeted by miR-145-5p, miR-101-3p and miR-335-5p and list of the relative publications

miR-145-5p			
SOX9	Zhuang, et al. 2022	FLI1	Chen et al., 2021
YES1	Sun et al., 2022	NF-κBp65	Tu et al., 2021
Smad3	Ye at al., 2022<br>Shen et al., 2020	CDCA3	Gu et al., 2021<br>Chen et al., 2021
ABRACL	Fan et al., 2021	SOCS7	Zeng et al., 2021
TMOD3	Li et al., 2021	KLF4	Tuo et al., 2021
IGF1R	Capik et al., 2021	RBBP5	Xie et al., 2021
SERPINE1	Li et al., 2021	S1PR1	Gao et al. 2021
CDH22	Zeng et al., 2021	CD36	Yuan et al., 2021
MYO6	Yang et al., 2021	Smad2	Shen et al., 2020
WIP1	Sun et al., 2021	HDAC11	Wang et al., 2020
NRAS	Yu et al., 2021<br>Ding et al., 2020<br>Li et al., 2020	CPEB4	Liu et al., 2020
MEST	Yu et al., 2021	ABCC1	Zheng et al., 2020
MORC2	Su et al., 2021	BACH2	Liu et al., 2020
BRD4	Wang et al, 2021	Smad4	Li et al., 2020<br>Liu et al., 2020<br>Zhou et al., 2020
SPOP	Zhang et al., 2021	ANGPT2	Zhou et al., 2020
TGF-β2	Qi et al., 2021	TNFAIP2	Li et al., 2020
PAK7	Chen et al., 2021	SPATS2	Dong et al., 2020
PXN	Lin et al, 2021	E2F3	Li et al., 2020
NOH-1	Tan et al., 2021	IGF1	Chen et al., 2020
AIFM1	Zhou et al., 2021	Notch1	Wei et al., 2020
NURR1	Jiang et al., 2021	ITGB8	Wei et al., 2020
CXCL1	Zhuang et al., 2021	CXCL3	Pei et al., 2020
ITGA2	Zhuang et al., 2021	CDK6	Shi et al., 2020<br>Shao et al., 2013
MUC1	Jara et al., 2021	SOX2	Tang et al., 2019
PLD5	Liu et al., 2021	FLT1	Lv et al., 2019
MAL2	He et al., 2021	KLF5	Dang et al., 2019<br>Zhou et al., 2019<br>Cao et al., 2020

ARF6	Zhong et al., 2021<br>Wang et al., 2021<br>Hsu et al., 2020	Sema3A	Liu et al., 2019
TOP2A	Du et al., 2021	TPT1	Jian et al., 2019<br>Du et al., 2019
Arhgap24	Zhang et al., 2021	RAB18	Wang et al., 2019
Srgap1	Zhang et al., 2021	RHBDD1	Niu et al., 2019
ROCK1	Cheng et al., 2021<br>Zhuang et al., 2021	FSCN1	Gao et al., 2019
SF-1	Liang et al., 2021	TGFbetaR2	García-García et al., 2019<br>Dong et al., 2021
FGF5	Zhu et al., 2021<br>Zhang et al., 2019	DUSP6	Wu et al., 2019<br>Gu et al., 2019
SOX4	Zhu et al., 2021	CDK6	Sun et al., 2019
TRPC6	Whang et al., 2021	MYD88	Sun et al., 2019
HMGA2	Tan et al., 2021	CTGF	Hang et al., 2019
TLR4	Jiang et al., 2021<br>Jin et al., 2019<br>Jara et al., 2021	PCBP2	Wang et al., 2019
PAFAH1B2	Xu et al., 2021	AKT3	Feng et al., 2019
VRK2	Mu et al., 2021	NEDD9	Yu et al., 2019
TRIM2	Xu et al., 2021	MTDH	Wang et al., 2015
TBX15	Zheng et al., 2021	SOX11	Chang et al., 2017
ATF3	Pan et al., 2021	ADAM17	Yang et al., 2014
EGFR	Cho et al., 2011	CFTR	Fabbri et al., 2017
ABRACL	Fan et al., 2021	JAM-A	Yang et al., 2020
CD40	Yuan et al., 2017	HBXIP	Jiang et al., 2019
BNIP3	Du et al., 2017	WNT2B	Li et al., 2019
UHRF1	Matsushita et al., 2016	AMAD19	Wang et al., 2019
FOXO1	Hao et al., 2018	REV3L	Chen et al., 2019
CCND2	Jin et al., 2020	EPHA4	Cai et al., 2019
TP53	Sheykhhasan et al., 2021	PAI-1	Liu et al., 2020
NUAK1	Xiong et al., 2018	TAGLN2	Zhang et al., 2018
STAT1	Gregersen et al., 2010	MRP1	Zhan et al., 2016
ADD3	Ye et al., 2017	PAK1	Kou et al., 2014
PDE8A	Booiman et al., 2014	ABCG2	Shi et al., 2014
DNMT3A	Li et al., 2020	TWIST	Shi et al., 2014
VEGFA	Zou et al., 2012	TNFRSF11B	Wang et al., 2017
HDAC2	Noh et al., 2013	LMNB2	Su et al., 2020

SP1	Zhu et al., 2014	p70S6K1	Xu et al., 2012
FKBP3	Zhu et al., 2017	OTC4	Hu et al., 2012
ERBB2	Sheykhhasan et al., 2021	HMGB3	Xie et al., 2020
PDGFRB	Xu et al., 2017	LRP5	Cai et al., 2020
MAPK1	Yang et al., 2018	LASP1	Wang et al., 2016
CBFB	Fukuda et al., 2015<br>Ostenfeld et al., 2010	SLC7A1	Wang et al., 2018
PPP3CA	Ostenfeld et al., 2010	C-MYC	Shao et al., 2013
CLINT1	Ostenfeld et al., 2010	SWAP70	Chiyomaru et al., 2011
IRS1	Guo et al., 2012	ESR1	Hu et al., 2018
NUDT1	Cho et al., 2011		

	**miR-335-5p**		
ARGLU1	Zhao et al., 2022	TPX2	Gu et al., 2020
TTK	Suyal et al., 2022	SNIP1	Xie et al., 2019
HBP1	Lu et al., 2021	IGF1R	Qi et al., 2019
MAPK10	Gao et al., 2021	LDHB	Zhang et al., 2019
SLC2A4	Li et al., 2021	ROCK1	Du et al., 2019
Oct4	Ji et al., 2021	OTC4	Yang et al., 2019
OCT4A	Taheri Bajgan et al., 2021	RB1	Shi et al., 2012
COL11A1	Kang et al ., 2021	ZEB2	Sun et al., 2014
CCNB2	Wang et al., 2020	SOX17	Yang et al., 2014
ADCY3	Zou al., 2020	RASA1	Wang et al., 2010
JNK3	Wang et al., 2020	BCL2L2	Liu et al., 2018
ROCK1	Li et al., 2020<br>She et al., 2020	ICAM1	Luo et al., 2018
SIX2	Jia et al., 2020	PYGO2	Qian et al., 2021
CUL4B	Yu et al., 2020	ZEB1	Zhang et al., 2018
NUCB2	Huo et al., 2020	CFTR	Song et al., 2021
CPNE1	Tang et al., 2018	CRIM1	Jiang et al., 2021
POU5F1	Gou et al., 2017	SP1	Wang et al., 2020
LRP1	Legaki et al., 2020	VEGF-C	Ping et al., 2021
SGK3	Yao et al., 2018	Tra2β	Liu et al., 2018
BIRC5	Zu et al., 2012	c-MET	Gao et al., 2015
TRIM29	Zhou et al., 2016	HIF1AN	Wu et al., 2018
c-MET	Gao et al., 2015	SGMS2	Pan et al., 2021

MAP3K2	Wang et al., 2021	NFIB	Lin et al., 2022
MEF2D	Wang et al., 2019	EphA4	Dong et al., 2018
EGR3	Zhang et al., 2019	E2F3	Song et al., 2021

**miR-101-3p**			
FOXP4	Xue et al., 2022	RAB5A	Sheng et al., 2014
HIPK3	Tao et al, 2022	KIF2A	Zhao et al., 2020
WEE1	Sun et al., 2022<br>Chen et al., 2019	SRF	Wu et al., 2017
USP47	Park et al., 2022	BICC1	Wang et al., 2020
BIRC5	Meng et al., 2021	SOX9	Liu et al., 2017
EZH2	Dong et al., 2021<br>Wang et al., 2018<br>Li et al., 2019	FOXP1	Sun et al., 2018
MAPK1	Zhao et al., 2021	PDCD4	Zhao et al., 2019
KPNA2	Wang et al., 2021	TLR2	Dong et al., 2015
DUSP1	Xin et al., 2021	APP	Barbato et al., 2019
SKP1	Zhang et al., 2021	RAC1	Lin et al., 2014
CUL4B	Gu et al., 2021<br>Zhang et al., 2019<br>Xie et al., 2021	IRF2BP2	Yao et al., 2019
MTOR	Gu et al., 2021	TRIB1	Niespolo et al., 2020
Notch1	Jiang et al., 2021	CMIP	Zhang et al., 2017
EIF4G2	Wang et al., 2021	CCN4	Pommier et al., 2021
VEGFA	Guo et al., 2021	MCL-1	Cui et al., 2018<br>Zhu et al., 2017
CLDN1	Du et al., 2021	COX2	Ma et al., 2016
TET2	Chen et al., 2020	ATP5B	Zheng et al., 2014
BICC1	Wang et al., 2020	CDH5	Cao et al., 2019
C/EBPα	Zhao et al., 2020	CPEB1	Xiaoping et al., 2013
KLF6	Zhao et al., 2020	ZEB2	Lin et al., 2019
STC1	An et al., 2020<br>Yuan et al., 2021	PIM1	Liu et al., 2015
KRAS	Ding et al., 2020	ATG4D	Frankel et al., 2011
PTGS2	Wei et al., 2020	RAB5A	Frankel et al., 2011
HDAC9	Sun et al., 2020	STMN1	Frankel et al., 2011<br>Zhu et al., 2018
RAP2B	Zhou et al., 2020	C-MET	Zhu et al., 2017
TRIM44	Li et al., 2019	HGF	Liu et al., 2020

ATX	Wang et al., 2019	NLK	Shen et al., 2014
MED19	Zhang et al., 2019	CFTR	Hassan et al., 2012
ZEB1	Fan et al., 2019	BICC1	Wang et al., 2020
Beclin-1	Sun et al., 2019	CXCR7	Yang et al., 2019
AMPK	Cao et al., 2019	MKP-1	Qiu et al., 2020
VEGF-C	Liu et al., 2019<br>Li et al., 2019	KDM1A	Huang et al., 2019
RAP1B	Zhou et al., 2020	KPNB1	Liu et al., 2019
		SOCS5	Zhang et al., 2020
CREB1	Yang et al., 2019	MEK1	Huang et al., 2019
HMGA2	Jiang et al., 2016	ANXA2	Bao et al., 2017
SOX4	Tavazoie et al., 2008	Nrf2	Dong et al., 2019
PTPRN2	Tavazoie et al., 2008	USP22	Zhao et al., 2016
MERTK	Tavazoie et al., 2008	FOXO1	Wang et al., 2017
TNC	Tavazoie et al., 2008	ABCC1	Shao etal., 2021
TIGAR	Xu et al., 2017	Jak2	Wang et al., 2014
CXCL12	Zhang et al., 2015	RAP1A	Chen et al., 2020
FOS	Liang et al 2014	TGF-β1	Wang et al., 2021
FZD4	Chen et al., 2019	RUNX1	Wang et al., 2015
ROCK2	Ye et al., 2016	PTGER4	Chandramouli et al., 2012

References

An, X.; Ma, H.; Liu, Y.; Li, F.; Song, Y.; Li, G.; Bai, Y.; Cao, B. Effects of miR-101-3p on goat granulosa cells in vitro and ovarian development in vivo via STC1. *J. Anim. Sci. Biotechnol.* **2020**, 14;11:102; Doi: 10.1186/s40104-020-00506-6.

Bao, J.; Xu, Y.; Wang, Q.; Zhang, J.; Li, Z.; Li, D.; Li, J. miR-101 alleviates chemoresistance of gastric cancer cells by targeting ANXA2. *Biomed. Pharmacother*. **2017**, 92:1030-1037; Doi: 10.1016/j.biopha.2017.06.011.

Barbato, C.; Pezzola, S.; Caggiano, C.; Antonelli, M.; Frisone, P.; Ciotti, M.T.; Ruberti, F. A lentiviral sponge for miR-101 regulates RanBP9 expression and amyloid precursor protein metabolism in hippocampal neurons. *Front. Cell Neurosci*. **2014**, 13;8:37; Doi: 10.3389/fncel.2014.00037.

Booiman, T.; Jiménez, V.C.; Dort, K.A.; Wout, A.B.; Kootstra, N.A. Phosphodiesterase 8a supports HIV-1 replication in macrophages at the level of reverse transcription. *PLoS One* **2014**, 9(10):e109673; Doi: 10.1371/journal.pone.0109673.

Cai, D.; Wei, D.; Chen, S.; Chen, X.; Li, S.; Chen, W.; He, W. MiR-145 protected the cell viability of human cerebral cortical neurons after oxygen-glucose deprivation by downregulating EPHA4. *Life Sci.* **2019**, 15;231:116517; Doi: 10.1016/j.lfs.2019.05.073.

Cai, W.L.; Zeng, W.; Liu, H.H.; Zhu, B.Y.; Liu, J.L.; Liu, Y. LncRNA LINC00707 promotes osteogenic differentiation of hBMSCs through the Wnt/β-catenin pathway activated by LINC00707/miR-145/LRP5 axis. *Eur. Rev. Med. Pharmacol Sci*. **2020**, 24(1):18-28; Doi: 10.26355/eurrev_202001_19891.

Cao, H.; Pan, G.; Tang, S.; Zhong, N.; Liu, H.; Zhou, H.; et al. miR-145-5p Regulates the Proliferation, Migration and Invasion in Cervical Carcinoma by Targeting KLF5. *Onco Targets Ther*. **2020**, 20;13:2369-2376; Doi: 10.2147/OTT.S241366.

Cao, S.; Li, L.; Geng, X.; Ma, Y.; Huang, X.; Kang, X. The upregulation of miR-101 promotes vascular endothelial cell apoptosis and suppresses cell migration in acute coronary syndrome by targeting CDH5. *Int. J. Clin. Exp. Pathol.* **2019**, 1;12(9):3320-3328.

Cao, S.; Lin, L.; Xia, X.; Wu, H. lncRNA SPRY4-IT1 Regulates Cell Proliferation and Migration by Sponging miR-101-3p and Regulating AMPK Expression in Gastric Cancer. *Mol. Ther. Nucleic. Acids*. **2019**, 6;17:455-464; Doi: 10.1016/j.omtn.2019.04.030.

Capik, O.; Sanli, F.; Kurt, A.; Ceylan, O.; Suer, I.; Kaya, M.; et al. CASC11 promotes aggressiveness of prostate cancer cells through miR-145/IGF1R axis. *Prostate Cancer Prostatic Dis*. **2021**, 24(3):891-902; Doi: 10.1038/s41391-021-00353-0.

Chandramouli, A.; Onyeagucha, B.C.; Mercado-Pimentel, M.E.; Stankova, L.; Shahin, N.A.; LaFleur, B.J.; et al. MicroRNA-101 (miR-101) post-transcriptionally regulates the expression of EP4 receptor in colon cancers. *Cancer Biol. Ther*. **2012**, 1;13(3):175-83; Doi: 10.4161/cbt.13.3.18874.

Chang, L.; Yuan, Z.; Shi, H.; Bian, Y.; Guo, R. miR-145 targets the SOX11 3'UTR to suppress endometrial cancer growth. *Am. J. Cancer Res*. **2017**, 1;7(11):2305-2317.

Chen, H.; Gao, J.; Xu, Q.; Wan, D.; Zhai, W.; Deng, L.; Qie, R. MiR-145-5p modulates lipid metabolism and M2 macrophage polarization by targeting PAK7 and regulating beta-catenin signaling in hyperlipidemia. *Can. J. Physiol. Pharmacol*. **2021**, 99(9):857-863; Doi: 10.1139/cjpp-2020-0539.

Chen, J.H.; Tong, W.; Pu, X.F.; Wang, J.Z. Long noncoding RNA CRNDE promotes proliferation, migration and invasion in prostate cancer through miR-101/Rap1A. *Neoplasma*. **2020**, 67(3):584-594; Doi: 10.4149/neo_2020_190621N534.

Chen, L.; Long, Y.; Han, Z.; Yuan, Z.; Liu, W.; Yang, F, et al. MicroRNA-101 inhibits cell migration and invasion in bladder cancer via targeting FZD4. *Exp. Ther. Med.* **2019**, 17(2):1476-1485; Doi: 10.3892/etm.2018.7084.

Chen, Q.; Hou, J.; Wu, Z.; Zhao, J.; Ma, D. miR-145 Regulates the sensitivity of esophageal squamous cell carcinoma cells to 5-FU via targeting REV3L. *Pathol. Res. Pract*. **2019**, 215(7):152427; Doi: 10.1016/j.prp.2019.04.019.

Chen, Q.; Li, X.; Kong, L.; Xu, Q.; Wang, Z.; Lv, Q. miR-101-3p induces vascular endothelial cell dysfunction by targeting tet methylcytosine dioxygenase 2. *Acta Biochim. Biophys Sin.* **2020**, 3;52(2):180-191; Doi: 10.1093/abbs/gmz154.

Chen, Q.; Zhou, L.; Ye, X.; Tao, M.; Wu, J. miR-145-5p suppresses proliferation, metastasis and EMT of colorectal cancer by targeting CDCA3. *Pathol. Res. Pract.* **2020**, 216(4):152872; Doi: 10.1016/j.prp.2020.152872.

Chen, X.; Zhang, N. Downregulation of lncRNA NEAT1_2 radiosensitizes hepatocellular carcinoma cells through regulation of miR-101-3p/WEE1 axis. *Cell Biol. Int*. **2019**, 43(1):44-55; Doi: 10.1002/cbin.11077.

Chen, Z.; Xu, W.; Zhang, D.; Chu, J.; Shen, S.; Ma, Y. circCAMSAP1 promotes osteosarcoma progression and metastasis by sponging miR-145-5p and regulating FLI1 expression. *Mol. Ther. Nucleic Acids.* **2020**, 23;23:1120-1135; Doi: 10.1016/j.omtn.2020.12.013.

Chen, Z.L.; Li, X.N.; Ye, C.X.; Chen, H.Y.; Wang, Z.J. Elevated Levels of circRUNX1 in Colorectal Cancer Promote Cell Growth and Metastasis via miR-145-5p/IGF1 Signalling. *Onco Targets Ther*. **2020**, 12;13:4035-4048; Doi: 10.2147/OTT.S254133.

Cheng, C.; Xu, D.L.; Liu, X.B.; Bi, S.J.; Zhang, J. MicroRNA-145-5p inhibits hypoxia/reoxygenation-induced apoptosis in H9c2 cardiomyocytes by targeting ROCK1. *Exp. Ther. Med*. **2021**, 22(2):796; Doi: 10.3892/etm.2021.10228.

Chiyomaru, T.; Tatarano, S.; Kawakami, K.; Enokida, H.; Yoshino, H.; Nohata, N.; et al. SWAP70, actin-binding protein, function as an oncogene targeting tumor-suppressive miR-145 in prostate cancer. *Prostate*. **2011**, 1;71(14):1559-67; Doi: 10.1002/pros.21372.

Cho, W.C.; Chow, A.S.; Au, J.S. MiR-145 inhibits cell proliferation of human lung adenocarcinoma by targeting EGFR and NUDT1. *RNA Biol.* **2011**, 8(1):125-31; Doi: 10.4161/rna.8.1.14259.

Cui, J.; Placzek, W.J. PTBP1 enhances miR-101-guided AGO2 targeting to MCL1 and promotes miR-101-induced apoptosis. *Cell Death Dis***. 2018**, 1;9(5):552; Doi: 10.1038/s41419-018-0551-8.

Dang, X.; Yang, L., Guo, J.; Hu, H.; Li, F.; Liu, Y.; Pang, Y. miR-145-5p is associated with smoke-related chronic obstructive pulmonary disease via targeting KLF5. *Chem. Biol. Interact*. **2019**, 25;300:82-90; Doi: 10.1016/j.cbi.2019.01.011.

Ding, B.; Fan, W.; Lou, W. hsa_circ_0001955 Enhances In Vitro Proliferation, Migration, and Invasion of HCC Cells through miR-145-5p/NRAS Axis. *Mol. Ther. Nucleic Acids.* **2020**, 16;22:445-455; Doi: 10.1016/j.omtn.2020.09.007.

Ding, C.; Xi, G.; Wang, G.; Cui, D.; Zhang, B.; Wang, H.; Jiang, G., Song, J.; Xu, G.; Wang, J. Exosomal Circ-MEMO1 Promotes the Progression and Aerobic Glycolysis of Non-small Cell Lung Cancer Through Targeting MiR-101-3p/KRAS Axis. *Front. Genet*. **2020**, 28;11:962; Doi: 10.3389/fgene.2020.00962.

Dong, G.; Zhang, S.; Shen, S.; Sun, L., Wang, X., Wang, H., et al. SPATS2, negatively regulated by miR-145-5p, promotes hepatocellular carcinoma progression through regulating cell cycle. *Cell Death Dis*. **2020**, 9;11(10):837; Doi: 10.1038/s41419-020-03039-y.

Dong, H.; Sun, Y.; Shan, F.; Sun, Q.; Yang, B. Down-Regulation of miR-101 Contributes to Rheumatic Heart Disease Through Up-Regulating TLR2. *Med. Sci. Monit*. **2015**, 25;21:1500-6; Doi: 10.12659/MSM.893540.

Dong, M.; Xu, T.; Li, H.; Li, X. LINC00052 promotes breast cancer cell progression and metastasis by sponging miR-145-5p to modulate TGFBR2 expression. *Oncol. Lett.* **2021**, 21(5):368; Doi: 10.3892/ol.2021.12629.

Dong, X.Q.; Zhang, Y.H.; Shang, X.Q.; Zeng, Y.J. Effects of miR-101 on the proliferation and apoptosis of gastric mucosal epithelial cells via Nrf2/ARE signaling pathway. *Eur. Rev. Med. Pharmacol. Sci*. **2019**, 23(12):5187-5194; Doi: 10.26355/eurrev_201906_18183.

Dong, Y.; Gao, Y.; Xie, T.; Liu, H.; Zhan, X.; Xu, Y. miR-101-3p Serves as a Tumor Suppressor for Renal Cell Carcinoma and Inhibits Its Invasion and Metastasis by Targeting EZH2. *Biomed. Res. Int*. **2021**, 7;2021:9950749; Doi: 10.1155/2021/9950749.

Dong, Y.; Liu, Y.; Jiang, A.; Li, R.; Yin, M.; Wang, Y. MicroRNA-335 suppresses the proliferation, migration, and invasion of breast cancer cells by targeting EphA4. *Mol. Cell Biochem*. **2018**, 439(1-2):95-104; Doi: 10.1007/s11010-017-3139-1.

Du, Q.; Hu, B.; Feng, Y.; Wang, Z.; Wang, X.; Zhu, D.; Zhu, Y.; Jiang, X.; Wang, H. circOMA1-Mediated miR-145-5p Suppresses Tumor Growth of Nonfunctioning Pituitary Adenomas by Targeting TPT1. *J. Clin. Endocrinol. Metab.* **2019**, 1;104(6):2419-2434; Doi: 10.1210/jc.2018-01851.

Du, W.; Tang, H.; Lei, Z.; Zhu, J.; Zeng, Y.; Liu, Z.; Huang, J.A. miR-335-5p inhibits TGF-beta1-induced epithelial-mesenchymal transition in non-small cell lung cancer via ROCK1. *Respir. Res*. **2019**, 21;20(1):225; Doi: 10.1186/s12931-019-1184-x.

Du, Y.; Li, J.; Xu, T.; Zhou, D.D.; Zhang, L.; Wang, X. MicroRNA-145 induces apoptosis of glioma cells by targeting BNIP3 and Notch signaling. *Oncotarget.* **2017**, 22;8(37):61510-61527; Doi: 10.18632/oncotarget.18604.

Du, Y.; Zhang, X.; Zhang, H.; Chen, Y.; Zhu, S.; Shu, J. et al. Propofol modulates the proliferation, invasion and migration of bladder cancer cells through the miR-145-5p/TOP2A axis. *Mol. Med. Rep*. **2021**, 23(6):439; Doi: 10.3892/mmr.2021.12078.

Du, Y.L.; Liang, Y.; Cao, Y.; Liu, L.; Li, J.; Shi, G.Q. LncRNA XIST Promotes Migration and Invasion of Papillary Thyroid Cancer Cell by Modulating MiR-101-3p/CLDN1 Axis. *Biochem. Genet*. **2021**, 59(2):437-452; Doi: 10.1007/s10528-020-09985-8.

Fabbri, E.; Tamanini, A.; Jakova, T.; Gasparello, J.; Manicardi, A.; Corradini, R.; et al. A Peptide Nucleic Acid against MicroRNA miR-145-5p Enhances the Expression of the Cystic Fibrosis Transmembrane Conductance Regulator (CFTR) in Calu-3 Cells. *Molecules.* **2017**, 29;23(1):71; Doi: 10.3390/molecules23010071.

Fan, M.J.; Zou, Y.H.; He, P.J.; Zhang, S.; Sun, X.M.; Li, C.Z. Long non-coding RNA SPRY4-IT1 promotes epithelial-mesenchymal transition of cervical cancer by regulating the miR-101-3p/ZEB1 axis. *Biosci Rep*. **2019**, 4;39(6):BSR20181339; Doi: 10.1042/BSR20181339.

Fan, S.; Chen, P.; Li, S. miR-145-5p inhibits the Proliferation, Migration, and Invasion of Esophageal Carcinoma Cells by Targeting ABRACL. *Biomed. Res. Int.* **2021**, 26;2021:6692544; Doi: 10.1155/2021/6692544.

Feng, J.; Zhou, Q.; Yi, H.; Ma, S.; Li, D.; Xu, Y.; Wang, J.; Yin, S. A novel lncRNA n384546 promotes thyroid papillary cancer progression and metastasis by acting as a competing endogenous RNA of miR-145-5p to regulate AKT3. *Cell Death Dis.* **2019**, 3;10(6):433; Doi: 10.1038/s41419-019-1637-7.

Frankel, L.B.; Wen, J.; Lees, M.; Høyer-Hansen, M.; Farkas, T.; Krogh, A.; et al. microRNA-101 is a potent inhibitor of autophagy. *EMBO J.* **2011**, 13;30(22):4628-41; Doi: 10.1038/emboj.2011.331.

Fukuda, T.; Ochi, H.; Sunamura, S.; Haiden, A.; Bando, W.; Inose, H.; et al. MicroRNA-145 regulates osteoblastic differentiation by targeting the transcription factor Cbfb. *FEBS Lett*. **2015**, 24;589(21):3302-8; Doi: 10.1016/j.febslet.2015.09.024.

Gao, W.; Zhang, C.; Li, W.; Li, H.; Sang, J.; Zhao, Q.; et al. Promoter Methylation-Regulated miR-145-5p Inhibits Laryngeal Squamous Cell Carcinoma Progression by Targeting FSCN1. *Mol. Ther.* **2019**, 6;27(2):365-379; Doi: 10.1016/j.ymthe.2018.09.018.

Gao, Y.; Ding, X. miR-145-5p exerts anti-tumor effects in diffuse large B-cell lymphoma by regulating S1PR1/STAT3/AKT pathway. *Leuk Lymphoma*. **2021**, 62(8):1884-1891; Doi: 10.1080/10428194.2021.1894642.

Gao, Y.; Wang, Y.; Wang, X.; Zhao, C.; Wang, F.; Du, J.; et al. miR-335-5p suppresses gastric cancer progression by targeting MAPK10. *Cancer Cell Int*. **2021**, 22;21(1):71; Doi: 10.1186/s12935-020-01684-z.

Gao, Y.; Zeng, F.; Wu, J.Y.; Li, H.Y.; Fan, J.J.; Mai, L.; Zhang, J.; Ma, D.M.; Li, Y.; Song, F.Z. MiR-335 inhibits migration of breast cancer cells through targeting oncoprotein c-Met. *Tumour Biol*. **2015**, 36(4):2875-83; Doi: 10.1007/s13277-014-2917-6.

García-García, F.; Salinas-Vera, Y.M.; García-Vázquez, R.; Marchat, L.A.; Rodríguez-Cuevas, S.; López-González, J.S.; et al. miR-145-5p is associated with pathological complete response to neoadjuvant chemotherapy and impairs cell proliferation by targeting TGFbetaR2 in breast cancer. *Oncol. Rep*. **2019**, 41(6):3527-3534; Doi: 10.3892/or.2019.7102.

Gregersen, L.H.; Jacobsen, A.B.; Frankel, L.B.; Wen, J.; Krogh, A.; Lund, A.H. MicroRNA-145 targets YES and STAT1 in colon cancer cells. *PLoS One*. **2010**, 21;5(1):e8836; Doi: 10.1371/journal.pone.0008836.

Gu, W.; Yuan, Y.; Wang, L.; Yang, H.; Li, S.; Tang, Z.; Li, Q. Long non-coding RNA TUG1 promotes airway remodelling by suppressing the miR-145-5p/DUSP6 axis in cigarette smoke-induced COPD. *J. Cell Mol. Med.* **2019**, 23(11):7200-7209; Doi: 10.1111/jcmm.14389.

Gu, X.; Yao, X.; Liu, D. Up-regulation of microRNA-335-5p reduces inflammation via negative regulation of the TPX2-mediated AKT/GSK3beta signaling pathway in a chronic rhinosinusitis mouse model. *Cell Signal*. **2020**, 70:109596; Doi: 10.1016/j.cellsig.2020.109596.

Gu, X.; Zhang, J.; Ran, Y.; Pan, H.; Jia, J.; Zhao, Y.; et al. Circular RNA hsa_circ_101555 promotes hepatocellular carcinoma cell proliferation and migration by sponging miR-145-5p and regulating CDCA3 expression. *Cell Death Dis*. **2021**, 6;12(4):356; Doi: 10.1038/s41419-021-03626-7.

Gu, Z.; You, Z.; Yang, Y.; Ding, R.; Wang, M.; Pu, J.; Chen, J. Inhibition of MicroRNA miR-101-3p on prostate cancer progression by regulating Cullin 4B (CUL4B) and PI3K/AKT/mTOR signaling pathways. *Bioengineered*. **2021**, 12(1):4719-4735; Doi: 10.1080/21655979.2021.1949513.

Guo, X.; Chen, M.; Cao, L.; Hu, Y.; Li, X.; Zhang, Q.; et al. Cancer-Associated Fibroblasts Promote Migration and Invasion of Non-Small Cell Lung Cancer Cells via miR-101-3p Mediated VEGFA Secretion and AKT/eNOS Pathway. *Front. Cell Dev. Biol*. **2021**, 16;9:764151; Doi: 10.3389/fcell.2021.764151.

Guo, X.; Yu, L.; Zhang, Z.; Dai, G.; Gao, T.; Guo, W. miR-335 negatively regulates osteosarcoma stem cell-like properties by targeting POU5F1. *Cancer Cell Int*. **2017**, 17;17:29; Doi: 10.1186/s12935-017-0398-6.

Guo, Y.; Chen, Y.; Zhang, Y.; Zhang, Y.; Chen, L.; Mo, D. Up-regulated miR-145 expression inhibits porcine preadipocytes differentiation by targeting IRS1. *Int. J. Biol. Sci*. **2012**, 8(10):1408-17; Doi: 10.7150/ijbs.4597.

Hang, W.; Feng, Y.; Sang, Z.; Yang, Y.; Zhu, Y.; Huang, Q.; Xi, X. Downregulation of miR-145-5p in cancer cells and their derived exosomes may contribute to the development of ovarian cancer by targeting CT. *Int. J. Mol. Med*. **2019**, 43(1):256-266; Doi: 10.3892/ijmm.2018.3958.

Hao, W.; Liu, H.; Zhou, L.; Sun, Y.; Su, H.; Ni, J.; He, T.; Shi, P.; Wang, X. MiR-145 regulates osteogenic differentiation of human adipose-derived mesenchymal stem cells through targeting FoxO1. *Exp. Biol. Med.* **2018**, 243(4):386-393; Doi: 10.1177/1535370217746611.

Hassan, F.; Nuovo, G.J.; Crawford, M.; Boyaka, P.N.; Kirkby, S.; Nana-Sinkam, S.P.; Cormet-Boyaka, E. MiR-101 and miR-144 regulate the expression of the CFTR chloride channel in the lung. *PLoS One*. **2012**, 7(11):e50837; Doi: 10.1371/journal.pone.0050837.

He, J.; Yan, H.; Wei, S.; Chen, G. LncRNA ST8SIA6-AS1 Promotes Cholangiocarcinoma Progression by Suppressing the miR-145-5p/MAL2 Axis. *Onco. Targets Ther.* **2021**, 17;14:3209-3223; Doi: 10.2147/OTT.S299634.

Hsu, W.C.; Li, W.M.; Lee, Y.C.; Huang, A.M.; Chang, L.L.; Lin, H.H.; et al. MicroRNA-145 suppresses cell migration and invasion in upper tract urothelial carcinoma by targeting ARF6. *FASEB J.* **2020**, 34(4):5975-5992; Doi: 10.1096/fj.201902555R.

Hu, H.B.; Chen, Q.; Ding, S.Q. LncRNA LINC01116 competes with miR-145 for the regulation of ESR1 expression in breast cancer. *Eur. Rev. Med. Pharmacol. Sci.* **2018**, 22(7):1987-1993; Doi: 10.26355/eurrev_201804_14726.

Hu, J.; Guo, H.; Li, H.; Liu, Y.; Liu, J.; Chen, L.; Zhang, J.; Zhang, N. MiR-145 regulates epithelial to mesenchymal transition of breast cancer cells by targeting Oct4. *PLoS One.* **2012**, 7(9):e45965; Doi: 10.1371/journal.pone.0045965.

Huang, Y.; Zou, Y.; Lin, L.; Ma, X.; Zheng, R. miR-101 regulates cell proliferation and apoptosis by targeting KDM1A in diffuse large B cell lymphoma. *Cancer Manag. Res.* **2019**, 5;11:2739-2746; Doi: 10.2147/CMAR.S197744.

Huang, Y.; Zou, Y.; Lin, L.; Ma, X.; Zheng, R. miR-101 regulates the cell proliferation and apoptosis in diffuse large B-cell lymphoma by targeting MEK1 via regulation of the ERK/MAPK signaling pathway. *Oncol. Rep.* **2019**, 41(1):377-386; Doi: 10.3892/or.2018.6821.

Huo, X.; Wang, H.; Huo, B.; Wang, L.; Yang, K.; Wang, J.; Wang, L.; Wang, H. FTX contributes to cell proliferation and migration in lung adenocarcinoma via targeting miR-335-5p/NUCB2 axis. *Cancer Cell Int.* **2020**, 23;20:89; Doi: 10.1186/s12935-020-1130-5.

Jara, D.; Carvajal, P.; Castro, I.; Barrera, M.J.; Aguilera, S.; González, S.; et al. Type I Interferon Dependent hsa-miR-145-5p Downregulation Modulates MUC1 and TLR4 Overexpression in Salivary Glands From Sjogren's Syndrome Patients. *Front. Immunol.* **2021**, 2;12:685837; Doi: 10.3389/fimmu.2021.685837.

Ji, Y.Y.; Song, Y.; Wang, A.N. MiR-335-5p inhibits proliferation of Huh-7 liver cancer cells via targeting the Oct4/Akt pathway. *Eur. Rev. Med. Pharmacol. Sci.* **2021**, 25(4):1853-1860; Doi: 10.26355/eurrev_202102_25080.

Jia, Q.; Ye, L.; Xu, S.; Xiao, H.; Xu, S.; Shi, Z.; Li, J.; Chen, Z. Circular RNA 0007255 regulates the progression of breast cancer through miR-335-5p/SIX2 axis. *Thorac. Cancer.* **2020**, 11(3):619-630; Doi: 10.1111/1759-7714.13306.

Jian, M.; Du, Q.; Zhu, D.; Mao, Z.; Wang, X.; Feng, Y.; et al. Tumor suppressor miR-145-5p sensitizes prolactinoma to bromocriptine by downregulating TPT1. *J. Endocrinol. Invest.* **2019**, 42(6):639-652; Doi: 10.1007/s40618-018-0963-4.

Jiang, L.; Li, M.; Gan, H.; Yang, L.; Lin, F.; Hu, M. miR-335 targets CRIM1 to promote the proliferation and inhibit the apoptosis of placental trophoblast cells in preeclamptic rats. *Am. J. Transl. Res.* **2021**, 13(5):4676-4685.

Jiang, L.; Wei, Z.C.; Xu, L.; Yu, S.Y.; Li, C. Inhibition of miR-145-5p Reduces Spinal Cord Injury-Induced Inflammatory and Oxidative Stress Responses via Affecting Nurr1-TNF-alpha Signaling Axis. *Cell Biochem. Biophys.* **2021**, 79(4):791-799; Doi: 10.1007/s12013-021-00992-z.

Jiang, R.; Zhang, Z.; Zhong, Z.; Zhang, C. Long-non-coding RNA RUSC1-AS1 accelerates osteosarcoma development by miR-101-3p-mediated Notch1 signalling pathway. *J. Bone Oncol*. **2021**, 16;30:100382; Doi: 10.1016/j.jbo.2021.100382.

Jiang, W.; Gu, W.; Qiu, R.; He, S.; Shen, C.; Wu, Y.; et al. miRNA-101 Suppresses Epithelial-to-Mesenchymal Transition by Targeting HMGA2 in Pancreatic Cancer Cells. *Anticancer Agents Med. Chem*. **2016**, 16(4):432-9; Doi: 10.2174/1871520615666150507122142.

Jiang, Y.; Wang, D.; Ren, H.; Shi, Y.; Gao, Y. MiR-145-targeted HBXIP modulates human breast cancer cell proliferation. *Thorac. Cancer*. **2019**, 10(1):71-77; Doi: 10.1111/1759-7714.12903.

Jiang, Z.; Zhang, J. Mesenchymal stem cell-derived exosomes containing miR-145-5p reduce inflammation in spinal cord injury by regulating the TLR4/NF-kappaB signaling pathway. *Cell Cycle*. **2021**, 20(10):993-1009; Doi: 10.1080/15384101.2021.1919825.

Jin, C.; Wang, A.; Liu, L.; Wang, G.; Li, G.; Han, Z. miR-145-5p inhibits tumor occurrence and metastasis through the NF-kappaB signaling pathway by targeting TLR4 in malignant melanoma. *J. Cell Biochem.* **2019**; Doi: 10.1002/jcb.28388.

Jin, M.; Ren, J.; Luo, M.; You, Z.; Fang, Y.; Han, Y.; et al. Long non-coding RNA JPX correlates with poor prognosis and tumor progression in non-small-cell lung cancer by interacting with miR-145-5p and CCND2. *Carcinogenesis*. **2020**, 10;41(5):634-645; Doi: 10.1093/carcin/bgz125.

Kang, Z.; Zhu, J.; Sun, N.; Zhang, X.; Liang, G.; Kou, Y.; et al. COL11A1 promotes esophageal squamous cell carcinoma proliferation and metastasis and is inversely regulated by miR-335-5p. *Ann. Transl. Med*. **2021**, 9(20):1577; Doi: 10.21037/atm-21-4951.

Kou, B.; Gao, Y.; Du, C.; Shi, Q.; Xu, S.; Wang, C.Q.; et al. miR-145 inhibits invasion of bladder cancer cells by targeting PAK1. *Urol. Oncol.* **2014**, 32(6):846-54; Doi: 10.1016/j.urolonc.2014.01.003.

Legaki, E.; Siasos, G.; Klonaris, C.; Athanasiadis, D.; Patelis, N.; Sioziou, A.; et al. Mir-335-5p as a potential regulator of LRP1 expression in abdominal aortic aneurysm. *Hellenic J. Cardiol*. **2020**, 61(6):430-432; Doi: 10.1016/j.hjc.2020.01.002.

Li, B.; Xie, D.; Zhang, H. MicroRNA-101-3p advances cisplatin sensitivity in bladder urothelial carcinoma through targeted silencing EZH2. *J. Cancer*. **2019**, 2;10(12):2628-2634; Doi: 10.7150/jca.33117.

Li, G.; Yang, F.; Gu, S.; Li, Z.; Xue, M. MicroRNA-101 induces apoptosis in cisplatin-resistant gastric cancer cells by targeting VEGF-C. *Mol. Med. Rep*. **2016**, 13(1):572-8; Doi: 10.3892/mmr.2015.4560.

Li, G.; Zhang, L. miR-335-5p aggravates type 2 diabetes by inhibiting SLC2A4 expression. *Biochem. Biophys Res. Commun*. **2021**, 18;558:71-78; Doi: 10.1016/j.bbrc.2021.04.011.

Li, H.; Pan, R.; Lu, Q.; Ren, C.; Sun, J.; Wu, H.; et al. MicroRNA-145-5p inhibits osteosarcoma cell proliferation by targeting E2F transcription factor 3. *Int. J. Mol. Med*. **2020**, 45(5):1317-1326; Doi: 10.3892/ijmm.2020.4504.

Li, H.; Zhao, S.; Chen, X.; Feng, G.; Chen, Z.; Fan, S. MiR-145 modulates the radiosensitivity of non-small cell lung cancer cells by suppression of TMOD3. *Carcinogenesis* **2021**, 9:bgab121; Doi: 10.1093/carcin/bgab121.

Li, H.W.; Liu, J. Circ_0009910 promotes proliferation and metastasis of hepatocellular carcinoma cells through miR-335-5p/ROCK1 axis. *Eur. Rev. Med. Pharmacol. Sci.* **2020**, 24(4):1725-1735; Doi: 10.26355/eurrev_202002_20349.

Li, J.; Song, Y.; Yu, B.; Yu, Y. TNFAIP2 Promotes Non-Small Cell Lung Cancer Cells and Targeted by miR-145-5p. *DNA Cell Biol*. **2020**, 39(7):1256-1263; Doi: 10.1089/dna.2020.5415.

Li, L.; Shao, M.Y.; Zou, S.C.; Xiao, Z.F.; Chen, Z.C. MiR-101-3p inhibits EMT to attenuate proliferation and metastasis in glioblastoma by targeting TRIM44. *J. Neurooncol*. **2019**, 141(1):19-30; Doi: 10.1007/s11060-018-2973-7.

Li, L.L.; Mao, C.D.; Wang, G.P.; Wang, N.; Xue, A.G. MiR-145-5p alleviates hypoxia/reoxygenation- induced cardiac microvascular endothelial cell injury in coronary heart disease by inhibiting Smad4 expression. *Eur. Rev. Med. Pharmacol. Sci.* **2020**, 24(9):5008-5017; Doi: 10.26355/eurrev_202005_21192.

Li, L.M.; Chen, C.; Ran, R.X.; Huang, J.T.; Sun, H.L.; Zeng, C.; et al. Loss of TARBP2 Drives the Progression of Hepatocellular Carcinoma via miR-145-SERPINE1 Axis. *Front. Oncol.* **2021**, 24;11:620912; Doi: 10.3389/fonc.2021.620912.

Li, Q.; Yu, X.; Yang, L. MiR-145 inhibits cervical cancer progression and metastasis by targeting WNT2B by Wnt/β-catenin pathway. *Int. J. Clin. Exp. Pathol*. **2019**, 1;12(10):3740-3751.

Li, X.; Liu, Y.; Zhang, X.; Shen, J.; Xu, R.; Liu, Y.; Yu, X. Circular RNA hsa_circ_0000073 contributes to osteosarcoma cell proliferation, migration, invasion and methotrexate resistance by sponging miR-145-5p and miR-151-3p and upregulating NRAS. *Aging* **2020**, 24;12(14):14157-14173; Doi: 10.18632/aging.103423.

Li, Y.; Jiang, B.; He, Z.; Zhu, H.; He, R.; Fan, S.; Wu, X.; Xie, L.; He, X. circIQCH sponges miR-145 to promote breast cancer progression by upregulating DNMT3A expression. *Aging* **2020**, 3;12(15):15532-15545; Doi: 10.18632/aging.103746.

Liang, J.; Li, H.; Mei, J.; Cao, Z.; Tang, Y.; Huang, R.; et al. Sertoli cell-derived exosome-mediated transfer of miR-145-5p inhibits Leydig cell steroidogenesis by targeting steroidogenic factor 1. *FASEB J.* **2021**, 35(6):e21660; Doi: 10.1096/fj.202002589RRRR.

Liang, X.; Liu, Y.; Zeng, L.; Yu, C.; Hu, Z.; Zhou, Q.; Yang, Z. miR-101 inhibits the G1-to-S phase transition of cervical cancer cells by targeting Fos. *Int. J. Gynecol. Cancer.* **2014**, 24(7):1165-72; Doi: 10.1097/IGC.0000000000000187.

Lin, D.; Wang, Y.; Lei, L.; Lin, C. Circ_0003645 serves as miR-335-5p sponge to promote the biological process of diffuse large B-cell lymphoma by upregulating NFIB. *Autoimmunity.* **2022** Mar, 55(2):127-135; Doi: 10.1080/08916934.2021.2023863.

Lin, H.; Zheng, X.; Lu, T.; Gu, Y.; Zheng, C.; Yan, H. The proliferation and invasion of osteosarcoma are inhibited by miR-101 via targetting ZEB2. *Biosci. Rep***. 2019**, 8;39(2):BSR20181283; Doi: 10.1042/BSR20181283.

Lin, K.H.; Kumar, V.B.; Shanmugam, T.; Shibu, M.A.; Chen, R.J.; Kuo, C.H.; et al. miR-145-5p targets paxillin to attenuate angiotensin II-induced pathological cardiac hypertrophy via downregulation of Rac 1, pJNK, p-c-Jun, NFATc3, ANP and by Sirt-1 upregulation. *Mol. Cell Biochem*. **2021**, 476(9):3253-3260; Doi: 10.1007/s11010-021-04100-w.

Lin, X.; Guan, H.; Li, H.; Liu, L.; Liu, J.; Wei, G.; Huang, Z.; Liao, Z.; Li, Y. miR-101 inhibits cell proliferation by targeting Rac1 in papillary thyroid carcinoma. *Biomed. Rep*. **2014**, 2(1):122-126; Doi: 10.3892/br.2013.192.

Liu, H.; Liu, P. Kartogenin Promotes the BMSCs Chondrogenic Differentiation in Osteoarthritis by Down-Regulation of miR-145-5p Targeting Smad4 Pathway. *Tissue Eng. Regen. Med*. **2021**, 18(6):989-1000; Doi: 10.1007/s13770-021-00390-9.

Liu, J.; Bian, T.; Feng, J.; Qian, L.; Zhang, J.; Jiang, D.; et al. miR-335 inhibited cell proliferation of lung cancer cells by target Tra2β. *Cancer Sci*. **2018**, 109(2):289-296; Doi: 10.1111/cas.13452.

Liu, J.; Li, J.; Ma, Y.; Xu, C.; Wang, Y.; He, Y. MicroRNA miR-145-5p inhibits Phospholipase D 5 (PLD5) to downregulate cell proliferation and metastasis to mitigate prostate cancer. *Bioengineered*. **2021**, 12(1):3240-3251; Doi: 10.1080/21655979.2021.1945361.

Liu, J.C.; Xue, D.F.; Wang, X.Q.; Ai, D.B.; Qin, P.J. MiR-101 relates to chronic peripheral neuropathic pain through targeting KPNB1 and regulating NF-κB signaling. *Kaohsiung J. Med. Sci*. **2019**, 35(3):139-145; Doi: 10.1002/kjm2.12025.

Liu, N.; Zhang, L.; Wang, Z.; Cheng, Y.; Zhang, P.; Wang, X.; et al. MicroRNA-101 inhibits proliferation, migration and invasion of human glioblastoma by targeting SOX9. *Oncotarget* **2017**, 21;8(12):19244-19254; Doi: 10.18632/oncotarget.13706.

Liu, P.; Li, X.; Cui, Y.; Chen, J.; Li, C.; Li, Q.; Li, H.; Zhang, X.; Zu, X. LncRNA-MALAT1 mediates cisplatin resistance via miR-101-3p/VEGF-C pathway in bladder cancer. *Acta Biochim. Biophys Sin*. **2019**, 18;51(11):1148-1157; Doi: 10.1093/abbs/gmz112.

Liu, R.; Guo, H.; Lu, S. MiR-335-5p restores cisplatin sensitivity in ovarian cancer cells through targeting BCL2L2. *Cancer Med*. **2018**, 7(9):4598-4609; Doi: 10.1002/cam4.1682.

Liu, X.; Su, K.; Kuang, S.; Fu, M.; Zhang, Z. miR-16-5p and miR-145-5p trigger apoptosis in human gingival epithelial cells by down-regulating BACH2. *Int. J. Clin. Exp. Pathol*. **2020**, 1;13(5):901-911.

Liu, X.; Zhao, H.; Li, W.; Bao, H.; Qu, Q.; Ma, D. Up-regulation of miR-145 may contribute to repeated implantation failure after IVF-embryo transfer by targeting PAI-1. *Reprod. Biomed. Online*. **2020**, 40(5):627-636; Doi: 10.1016/j.rbmo.2020.01.018.

Liu, X.; Zhu, W.; Wang, L.; Wu, J.; Ding, F.; Song, Y. miR-145-5p suppresses osteogenic differentiation of adipose-derived stem cells by targeting semaphorin 3A. *In Vitro Cell Dev. Biol. Anim*. **2019**, 55(3):189-202; Doi: 10.1007/s11626-019-00318-7.

Liu, X.Y.; Liu, Z.J.; He, H.; Zhang, C.; Wang, Y.L. MicroRNA-101-3p suppresses cell proliferation, invasion and enhances chemotherapeutic sensitivity in salivary gland adenoid cystic carcinoma by targeting Pim-1. *Am. J. Cancer Res*. **2015**, 15;5(10):3015-29.

Liu, Y.; Li ,R.; Wang, X.; Yang, W. CircTTBK2 Contributes to the Progression of Glioma Through Regulating miR-145-5p/CPEB4 Axis. *Cancer Manag. Res*. **2020**, 8;12:8183-8195; Doi: 10.2147/CMAR.S263586.

Liu, Y.; Tan, J.; Ou, S.; Chen, J.; Chen, L. MicroRNA-101-3p suppresses proliferation and migration in hepatocellular carcinoma by targeting the HGF/c-Met pathway. *Invest. New Drugs*. **2020**, 38(1):60-69; Doi: 10.1007/s10637-019-00766-8.

Lu, X.; Li, Y.; Chen, H.; Pan, Y.; Lin, R.; Chen, S. miR-335-5P contributes to human osteoarthritis by targeting HBP1. *Exp. Ther. Med*. **2021**, 21(2):109; Doi: 10.3892/etm.2020.9541.

Luo, L.; Xia, L.; Zha, B.; Zuo, C.; Deng, D.; Chen, M.; et al. miR-335-5p targeting ICAM-1 inhibits invasion and metastasis of thyroid cancer cells. *Biomed. Pharmacother.* **2018**, 106:983-990; Doi: 10.1016/j.biopha.2018.07.046.

Lv, Y.; Lu, X.; Li, C.; Fan, Y.; Ji, X.; Long, W.; Meng, L.; Wu, L.; Wang, L.; Lv, M.; Ding, H. miR-145-5p promotes trophoblast cell growth and invasion by targeting FLT1. *Life Sci.* **2019**, 15;239:117008; Doi: 10.1016/j.lfs.2019.117008.

Ma, C.; Zheng, C.; Bai, E.; Yang, K. miR-101 inhibits glioma cell invasion via the downregulation of COX-2. *Oncol Lett*. **2016**, 12(4):2538-2544; Doi: 10.3892/ol.2016.4939.

Matsushita, R.; Yoshino, H.; Enokida, H.; Goto, Y.; Miyamoto, K.; Yonemori, M.; et al. Regulation of UHRF1 by dual-strand tumor-suppressor microRNA-145 (miR-145-5p and miR-145-3p): Inhibition of bladder cancer cell aggressiveness. *Oncotarget.* **2016**, 10;7(19):28460-87; Doi: 10.18632/oncotarget.8668.

Meng, X.; Sun, Y.; Liu, S.; Mu, Y. miR-101-3p sensitizes lung adenocarcinoma cells to irradiation via targeting BIRC5. *Oncol Lett.* **2021**, 21(4):282; Doi: 10.3892/ol.2021.12543.

Mu, Y.; Liu, W.J.; Bie, L.Y.; Mu, X.Q.; Zhao, Y.Q. Blocking VRK2 suppresses pulmonary adenocarcinoma progression via ERK1/2/AKT signal pathway by targeting miR-145-5p. *Eur. Rev. Med. Pharmacol. Sci*. **2021**, 25(1):145-153; Doi: 10.26355/eurrev_202101_24378.

Niespolo, C.; Johnston, J.M.; Deshmukh, S.R.; Satam, S.; Shologu, Z.; Villacanas, O.; et al. Tribbles-1 Expression and Its Function to Control Inflammatory Cytokines, Including Interleukin-8 Levels are Regulated by miRNAs in Macrophages and Prostate Cancer Cells. *Front. Immunol.* **2020**, 27;11:574046; Doi: 10.3389/fimmu.2020.574046.

Niu, Y.; Zhang, J.; Tong, Y.; Li, J.; Liu, B. miR-145-5p restrained cell growth, invasion, migration and tumorigenesis via modulating RHBDD1 in colorectal cancer via the EGFR-associated signaling pathway. *Int. J. Biochem. Cell Biol.* **2019**, 117:105641; Doi: 10.1016/j.biocel.2019.105641.

Noh, J.H.; Chang, Y.G.; Kim, M.G.; Jung, K.H.; Kim, J.K.; Bae, H.J.; et al. MiR-145 functions as a tumor suppressor by directly targeting histone deacetylase 2 in liver cancer. *Cancer Lett.* **2013**, 28;335(2):455-62; Doi: 10.1016/j.canlet.2013.03.003.

Ostenfeld, M.S.; Bramsen, J.B.; Lamy, P.; Villadsen, S.B.; Fristrup, N.; Sørensen, K.D.; et al. miR-145 induces caspase-dependent and -independent cell death in urothelial cancer cell lines with targeting of an expression signature present in Ta bladder tumors. *Oncogene* **2010**, 18;29(7):1073-84; Doi: 10.1038/onc.2009.395.

Pan, J.; Xu, Z.; Guo, G.; Xu, C.; Song, Z.; Li, K.; et al. Circ_nuclear factor I X (circNfix) attenuates pressure overload-induced cardiac hypertrophy via regulating miR-145-5p/ATF3 axis. *Bioengineered*. **2021**, 12(1):5373-5385; Doi: 10.1080/21655979.2021.1960462.

Pan, Q.; Li, B.; Zhang, J.; Du, X.; Gu, D. LncRNA THAP9-AS1 accelerates cell growth of esophageal squamous cell carcinoma through sponging miR-335-5p to regulate SGMS2. *Pathol. Res. Pract*. **2021**, 224:153526; Doi: 10.1016/j.prp.2021.153526.

Park, J.; Cho, M.; Cho, J.; Kim, E.E.; Song, E.J. MicroRNA-101-3p Suppresses Cancer Cell Growth by Inhibiting the USP47-Induced Deubiquitination of RPL11. *Cancers* **2022**, 15;14(4):964; Doi: 10.3390/cancers14040964.

Pei, X.; Chen, S.W.; Long, X.; Zhu, S.Q.; Qiu, B.Q.; Lin, K. et al. circMET promotes NSCLC cell proliferation, metastasis, and immune evasion by regulating the miR-145-5p/CXCL3 axis. *Aging*. **2020**, 2;12(13):13038-13058; Doi: 10.18632/aging.103392.

Ping, Q.; Shi, Y.; Yang, M.; Li, H.; Zhong, Y.; Li, J.; Bi, X.; Wang, C. LncRNA DANCR regulates lymphatic metastasis of bladder cancer via the miR-335/VEGF-C axis. *Transl. Androl. Urol*. **2021**, 10(4):1743-1753; Doi: 10.21037/tau-21-226.

Pommier, A.; Varilh, J.; Bleuse, S.; Delétang, K.; Bonini, J.; Bergougnoux, A.; et al. miRNA repertoires of cystic fibrosis ex vivo models highlight miR-181a and miR-101 that regulate WISP1 expression. *J. Pathol.* **2021**, 253(2):186-197; Doi: 10.1002/path.5571.

Qi, J.; Shi, L.Y.; Wu, Y.; Shen, X.J.; Yuan, J.; Jin, C.J.; Cong, H.; Ju, S.Q. Epigenetic silencing of miR-335 induces migration by targeting insulin-like growth factor-1 receptor in multiple myeloma. *Leuk. Lymphoma***. 2019**, 60(13):3188-3198; Doi: 10.1080/10428194.2019.1627534.

Qi, X.; Liu, Y.; Yang, M. Circ_0057452 functions as a ceRNA in hypertrophic scar fibroblast proliferation and VEGF expression by regulating TGF-beta2 expression and adsorbing miR-145-5p. *Am. J. Transl Res*. **2021**, 15;13(6):6200-6210.

Qian, J.; Lei, X.; Sun, Y.; Zheng, L.; Li, J.; Zhang, S. Long non-coding RNA SNHG8 enhances triple-negative breast cancer cell proliferation and migration by regulating the miR-335-5p/PYGO2 axis. *Biol. Direct.* **2021**, 6;16(1):13; Doi: 10.1186/s13062-021-00295-6.

Qiu, S.; Liu, B.; Mo, Y.; Wang, X.; Zhong, L.; Han, X.; Mi, F. MiR-101 promotes pain hypersensitivity in rats with chronic constriction injury via the MKP-1 mediated MAPK pathway. *J. Cell. Mol. Med*. **2020**, 24(16):8986-8997; Doi: 10.1111/jcmm.15532.

Shao, N.; Song, L.; Sun, X. Exosomal circ_PIP5K1A regulates the progression of non-small cell lung cancer and cisplatin sensitivity by miR-101/ABCC1 axis. *Mol. Cell. Biochem*. **2021**, 476(6):2253-2267; Doi: 10.1007/s11010-021-04083-8.

Shao, Y.; Qu, Y.; Dang, S.; Yao, B.; Ji, M. MiR-145 inhibits oral squamous cell carcinoma (OSCC) cell growth by targeting c-Myc and Cdk6. *Cancer Cell Int*. **2013**, 28;13(1):51; Doi: 10.1186/1475-2867-13-51.

She, J.K.; Fu, D.N.; Zhen, D.; Gong, G.H.; Zhang, B. LINC01087 is Highly Expressed in Breast Cancer and Regulates the Malignant Behavior of Cancer Cells Through miR-335-5p/Rock1. *Onco. Targets Ther*. **2020**, 30;13:9771-9783; Doi: 10.2147/OTT.S255994.

Shen, Q.; Bae, H.J.; Eun, W.J.; Kim, H.S.; Park, S.J.; Shin, W.C. MiR-101 functions as a tumor suppressor by directly targeting nemo-like kinase in liver cancer. *Cancer Lett*. **2014**, 28;344(2):204-11; Doi: 10.1016/j.canlet.2013.10.030.

Shen, W.; Wang, Y.; Wang, D.; Zhou, H.; Zhang, H.; Li, L. miR-145-5p attenuates hypertrophic scar via reducing Smad2/Smad3 expression. *Biochem. Biophys. Res Commun.* **2020**, 22;521(4):1042-1048; Doi: 10.1016/j.bbrc.2019.11.040.

Shen, X.; Jiang, H.; Chen, Z.; Lu, B.; Zhu, Y.; Mao, J.; Chai, K.; Chen, W. MicroRNA-145 Inhibits Cell Migration and Invasion in Colorectal Cancer by Targeting TWIST. *Onco. Targets Ther*. **2019**, 10;12:10799-10809; Doi: 10.2147/OTT.S216147.

Sheng, Y.; Li, J.; Zou, C.; Wang, S.; Cao, Y.; Zhang, J.; Huang, A.; Tang, H. Downregulation of miR-101-3p by hepatitis B virus promotes proliferation and migration of hepatocellular carcinoma cells by targeting Rab5a. *Arch. Virol*. **2014**, 159(9):2397-410; Doi: 10.1007/s00705-014-2084-5.

Sheykhhasan, M.; Kalhor, N.; Sheikholeslami, A.; Dolati, M.; Amini, E.; Fazaeli, H. Exosomes of Mesenchymal Stem Cells as a Proper Vehicle for Transfecting miR-145 into the Breast Cancer Cell Line and Its Effect on Metastasis. *Biomed. Res. Int.* **2021**, 29;2021:5516078; Doi: 10.1155/2021/5516078.

Shi, L.; Jiang, D.; Sun, G.; Wan, Y.; Zhang, S.; Zeng, Y.; Pan, T.; Wang, Z. miR-335 promotes cell proliferation by directly targeting Rb1 in meningiomas. *J. Neurooncol.* **2012**, 110(2):155-62; Doi: 10.1007/s11060-012-0951-z.

Shi, L.; Wang, Z.; Sun, G.; Wan, Y.; Guo, J.; Fu, X. miR-145 inhibits migration and invasion of glioma stem cells by targeting ABCG2. *Neuromolecular Med.* **2014**, 16(2):517-28; Doi: 10.1007/s12017-014-8305-y.

Shi, W.; Gao, Z.; Song, J.; Wang, W. Silence of FOXD2-AS1 inhibited the proliferation and invasion of esophagus cells by regulating miR-145-5p/CDK6 axis. *Histol. Histopathol.* **2020**, 35(9):1013-1021; Doi: 10.14670/HH-18-232.

Song, J.; Zhang, Z. Long non-coding RNA SNHG20 promotes cell proliferation, migration and invasion in retinoblastoma via the miR-335-5p/E2F3 axis. *Mol. Med. Rep.* **2021**, 24(2):543; Doi: 10.3892/mmr.2021.12182.

Su, X.; Feng, X.; Gao, C.; Wang, G.; Liu, L. ROR promotes the proliferation and migration of esophageal cancer through regulating miR-145/LMNB2 signal axis. *Am. J. Transl. Res.* **2020**, 15;12(11):7223-7235.

Su, Y.; Yu, T.; Wang, Y.; Huang, X.; Wei, X. Circular RNA circDNM3OS Functions as a miR-145-5p Sponge to Accelerate Cholangiocarcinoma Growth and Glutamine Metabolism by Upregulating MORC2. *Onco Targets Ther.* **2021**, 17;14:1117-1129; Doi: 10.2147/OTT.S289241.

Sun, H.; Guo, F.; Xu, L. Downregulation of microRNA-101-3p participates in systemic lupus erythematosus progression via negatively regulating HDAC9. *J. Cell. Biochem.* **2020**, 121(10):4310-4320; Doi: 10.1002/jcb.29624.

Sun, J.; Deng, L.; Gong, Y.J. MiR-145-5p Inhibits the Invasion of Prostate Cancer and Induces Apoptosis by Inhibiting WIP1. *Oncol.* **2021**, 2;2021:4412705; Doi: 10.1155/2021/4412705.

Sun, L.; Cai, H.; Zhou, T.; Xiang, H.; Long, L.; et al. Verbascoside enhances radiosensitivity of hepatocellular carcinoma cells through regulating miR-101-3p/Wee1 axis. *Drug Dev. Res.* **2022**, 26; Doi: 10.1002/ddr.21914.

Sun, M.; Zhao, W.; Chen, Z.; Li, M.; Li, S.; Wu, B.; Bu, R. Circ_0058063 regulates CDK6 to promote bladder cancer progression by sponging miR-145-5p. *J. Cell. Physiol.* **2019**, 234(4):4812-4824; Doi: 10.1002/jcp.27280.

Sun, M.; Zhao, W.; Chen, Z.; Li, M.; Li, S.; Wu, B.; Bu, R. Circular RNA CEP128 promotes bladder cancer progression by regulating Mir-145-5p/Myd88 via MAPK signaling pathway. *Int. J. Cancer.* **2019**, 15;145(8):2170-2181; Doi: 10.1002/ijc.32311.

Sun, W.; Zhang, Q.; Wu, Z.; Xue, N. miR-101-3p sensitizes hepatocellular carcinoma cells to oxaliplatin by inhibiting Beclin-1-mediated autophagy. *Int. J. Clin. Exp. Pathol.* **2019**, 1;12(6):2056-2065.

Sun, Y.; Liu, J.; Chu, L.; Yang, W.; Liu, H.; Li, C.; Yang, J. Long noncoding RNA SNHG12 facilitates the tumorigenesis of glioma through miR-101-3p/FOXP1 axis. *Gene* **2018**, 15;676:315-321; Doi: 10.1016/j.gene.2018.08.034.

Sun, Y.; Tian, Y.; He, J.; Tian, Y.; Zhang, G.; Zhao, R, et al. Linc01133 contributes to gastric cancer growth by enhancing YES1-dependent YAP1 nuclear translocation via sponging miR-145-5p. *Cell Death Dis.* **2022**, 13(1):51; Doi: 10.1038/s41419-022-04500-w.

Sun, Z.; Zhang, Z.; Liu, Z.; Qiu, B.; Liu, K.; Dong, G. MicroRNA-335 inhibits invasion and metastasis of colorectal cancer by targeting ZEB2. *Med. Oncol*. **2014**, 31(6):982; Doi: 10.1007/s12032-014-0982-8.

Suyal, G.; Pandey, P.; Saraya, A.; Sharma, R. Tumour suppressor role of microRNA-335-5p in esophageal squamous cell carcinoma by targeting TTK (Mps1). *Exp. Mol. Pathol*. **2022**, 124:104738; Doi: 10.1016/j.yexmp.2021.104738.

Taheri Bajgan, E.; Gholipour, A.; Faghihi, M.; Mowla, S.J.; Malakootian, M. Linc-ROR has a Potential ceRNA Activity for OCT4A by Sequestering miR-335-5p in the HEK293T Cell Line. *Biochem. Genet.* **2021**; Doi: 10.1007/s10528-021-10140-0.

Tamanini, A.; Fabbri, E.; Jakova, T.; Gasparello, J.; Manicardi, A.; Corradini, R.; et al. A Peptide-Nucleic Acid Targeting miR-335-5p Enhances Expression of Cystic Fibrosis Transmembrane Conductance Regulator (CFTR) Gene with the Possible Involvement of the CFTR Scaffolding Protein NHERF1. *Biomedicines*. **2021**, 26;9(2):117; Doi: 10.3390/biomedicines9020117.

Tan, L.; Liu, L.; Yao, J.; Piao, C. miR-145-5p attenuates inflammatory response and apoptosis in myocardial ischemia-reperfusion injury by inhibiting (NADPH) oxidase homolog 1. *Exp. Anim*. **2021**, 6;70(3):311-321; Doi: 10.1538/expanim.20-0160.

Tan, S.; Chen, J. si-MALAT1 attenuates thymic cancer cell proliferation and promotes apoptosis via the miR-145-5p/HMGA2 pathway. *Oncol. Lett*. **2021**, 22(2):585; Doi: 10.3892/ol.2021.12846.

Tang, H.; Zhu, J.; Du, W.; Liu, S.; Zeng, Y.; Ding, Z.; et al. CPNE1 is a target of miR-335-5p and plays an important role in the pathogenesis of non-small cell lung cancer. *J. Exp. Clin. Cancer Res*. **2018**, 3;37(1):131.

Tang, W.; Zhang, X.; Tan, W.; Gao, J.; Pan, L.; Ye, X.; et al. miR-145-5p Suppresses Breast Cancer Progression by Inhibiting SOX2. *J. Surg. Res*. **2019**, 236:278-287; Doi: 10.1016/j.jss.2018.11.030.

Tao, L.; Xu, C.; Shen, W.; Tan, J.; Li, L.; Fan, M.; et al. HIPK3 Inhibition by Exosomal hsa-miR-101-3p Is Related to Metabolic Reprogramming in Colorectal Cancer. *Front. Oncol.* **2022**, 13;11:758336; Doi: 10.3389/fonc.2021.758336.

Tavazoie, S.F.; Alarcón, C.; Oskarsson, T.; Padua, D.; Wang, Q.; Bos, P.D.; et al. Endogenous human microRNAs that suppress breast cancer metastasis. *Nature* **2008**, 10; 451(7175): 147–152; Doi: 10.1038/nature06487.

Tu, Y.H.; Guo, Y.; Ji, S.; Shen, J.L.; Fei, G.H.; et al. The Influenza A Virus H3N2 Triggers the Hypersusceptibility of Airway Inflammatory Response via Activating the lncRNA TUG1/miR-145-5p/NF-kappaB Pathway in COPD. *Front. Pharmacol.* **2021**, 22;12:604590; Doi: 10.3389/fphar.2021.604590.

Tuo, Z.; Liang, L.; Zhou, R. LINC00852 is associated with poor prognosis in non-small cell lung cancer patients and its inhibition suppresses cancer cell proliferation and chemoresistance via the hsa-miR-145-5p/KLF4 axis. *J. Gene Med.* **2021**, 23(12):e3384; Doi: 10.1002/jgm.3384.

Wang, A.D.; Dai, L.F.; Yang, L.; Wang, Y.S.; Hao, X.H.; Liu, Z.C.; Chen, P.L. Upregulation of miR-335 reduces myocardial injury following myocardial infarction via targeting MAP3K2. *Eur. Rev. Med. Pharmacol. Sci*. **2021**, 25(1):344-352; Doi: 10.26355/eurrev_202101_24401.

Wang, B.; Dong, W.; Li, X. miR-145-5p Acts as a Novel Tumor Suppressor in Hepatocellular Carcinoma Through Targeting RAB18. *Technol. Cancer Res. Treat*. **2019**, 1;18:1533033819850189; Doi: 10.1177/1533033819850189.

Wang, C.; Liu, B. miR-101-3p induces autophagy in endometrial carcinoma cells by targeting EZH2. *Arch. Gynecol Obstet.* **2018**, 297(6):1539-1548; Doi: 10.1007/s00404-018-4768-7.

Wang, D.; Fei, Z.; Luo, S.; Wang, H. MiR-335-5p Inhibits beta-Amyloid (Abeta) Accumulation to Attenuate Cognitive Deficits Through Targeting c-jun-N-terminal Kinase 3 in Alzheimer's Disease. *Curr. Neurovasc. Res.* **2020**, 17(1):93-101; Doi: 10.2174/1567202617666200128141938.

Wang, G.D.; Zhao, X.W.; Zhang, Y.G.; Kong, Y.; Niu, S.S.; Ma, L.F.; Zhang, Y.M. Effects of miR-145 on the inhibition of chondrocyte proliferation and fibrosis by targeting TNFRSF11B in human osteoarthritis. *Mol. Med. Rep*. **2017**, 15(1):75-80; Doi: 10.3892/mmr.2016.5981.

Wang, H.; Guo, Y.; Mi, N.; Zhou, L. miR-101-3p and miR-199b-5p promote cell apoptosis in oral cancer by targeting BICC1. *Mol. Cell. Probes*. **2020**, 52:101567; Doi: 10.1016/j.mcp.2020.101567.

Wang, H.; Xiao, R.; Yang, B. MiR-101-3p Suppresses Progression of Cervical Squamous Cell Carcinoma by Targeting and Down-Regulating KPNA2. *Technol. Cancer Res. Treat*. **2021**, 20:15330338211055948; Doi: 10.1177/15330338211055948.

Wang, J.; Ruan, K. miR-335 is involved in the rat epididymal development by targeting the mRNA of RASA1. *Biochem. Biophys Res. Commun*. **2010**, 12;402(2):222-7; Doi: 10.1016/j.bbrc.2010.10.002.

Wang, J.; Zhang, H.; Situ, J.; Li, M.; Sun, H. KCNQ1OT1 aggravates cell proliferation and migration in bladder cancer through modulating miR-145-5p/PCBP2 axis. *Cancer Cell Int.* **2019**, 3;19:325; Doi: 10.1186/s12935-019-1039-z.

Wang, L.; Li, L.; Guo, R.; Li, X.; Lu, Y.; Guan, X.; et al. miR-101 promotes breast cancer cell apoptosis by targeting Janus kinase 2. *Cell Physiol. Biochem***.** **2014**, 34(2):413-22; Doi: 10.1159/000363010.

Wang, M.; Wang, J.; Deng, J.; Li, X.; Long, W.; Chang, Y. MiR-145 acts as a metastasis suppressor by targeting metadherin in lung cancer. *Med. Oncol.* **2015**, 32(1):344; Doi: 10.1007/s12032-014-0344-6.

Wang, Q.; Tao, Y.; Xie, H.; Liu, C.; Liu, P. MicroRNA-101 inhibits renal tubular epithelial-to-mesenchymal transition by targeting TGF-β1 type I receptor. *Int. J. Mol. Med*. **2021**, 47(6):119; Doi: 10.3892/ijmm.2021.4952.

Wang, S.; Li, Y.; Sun, S.; Cai, J.; Cao, J. Sp1 promotes ovarian cancer cell migration through repressing miR-335 expression. *Biochem. Biophys Res. Commun*. **2020**, 26;524(1):211-216; Doi: 10.1016/j.bbrc.2020.01.063.

Wang, S.; Wang, T.; Gu, P. microRNA-145-5p Inhibits Migration, Invasion, and Metastasis in Hepatocellular Carcinoma by Inhibiting ARF6. *Cancer Manag. Res*. **2021**, 21;13:3473-3484; Doi: 10.2147/CMAR.S300678.

Wang, W.; Chen, L.C.; Qian, J.Y.; Zhang, Q. MiR-335 promotes cell proliferation by inhibiting MEF2D and sensitizes cells to 5-Fu treatment in gallbladder carcinoma. *Eur. Rev. Med. Pharmacol. Sci*. **2019**, 23(22):9829-9839; Doi: 10.26355/eurrev_201911_19546.

Wang, W.; Ding, B.; Lou, W.; Lin, S. Promoter Hypomethylation and miR-145-5p Downregulation- Mediated HDAC11 Overexpression Promotes Sorafenib Resistance and Metastasis of Hepatocellular Carcinoma Cells. *Front. Cell Dev. Biol.* **2020**, 12;8:724; Doi: 10.3389/fcell.2020.00724.

Wang, W.; Ji, G.; Xiao, X.; Chen, X.; Qin, W.W.; Yang, F.; et al. Epigenetically regulated miR-145 suppresses colon cancer invasion and metastasis by targeting LASP1. *Oncotarget.* **2016**, 18;7(42):68674-68687; Doi: 10.18632/oncotarget.11919.

Wang, X.; Bai, X.; Yan, Z.; Guo, X.; Zhang, Y. The lncRNA TUG1 promotes cell growth and migration in colorectal cancer via the TUG1-miR-145-5p-TRPC6 pathway. *Biochem. Cell Biol*. **2021**, 99(2):249-260; Doi: 10.1139/bcb-2020-0017.

Wang, X.; Xiao, H.; Wu, D.; Zhang, D.; Zhang, Z. miR-335-5p Regulates Cell Cycle and Metastasis in Lung Adenocarcinoma by Targeting CCNB2. *Onco. Targets Ther*. **2020**, 30;13:6255-6263; Doi: 10.2147/OTT.S245136.

Wang, X.; Zhao, Y.; Qian, H.; Huang, J.; Cui, F.; Mao, Z. The miR-101/RUNX1 feedback regulatory loop modulates chemo-sensitivity and invasion in human lung cancer. *Int. J. Clin. Exp. Med*. **2015**, 15;8(9):15030-42.

Wang, Y.; Jin, L. miRNA-145 is associated with spontaneous hypertension by targeting SLC7A1. *Exp. Ther. Med.* **2018**, 15(1):548-552; Doi: 10.3892/etm.2017.5371.

Wang, Y.; Lian, Y.M.; Ge, C.Y. MiR-145 changes sensitivity of non-small cell lung cancer to gefitinib through targeting ADAM19. *Eur. Rev. Med. Pharmacol. Sci.* **2019**, 23(13):5831-5839; Doi: 10.26355/eurrev_201907_18323.

Wang, Y.; Lyu, L.; Zhang, X.; Zhang, J. Autotaxin is a novel target of microRNA-101-3p. *FEBS Open Bio*. **2019**, 1;9(4):707-716; Doi: 10.1002/2211-5463.12608.

Wang, Y.; Tian, H. miR-101 suppresses HBV replication and expression by targeting FOXO1 in hepatoma carcinoma cell lines. *Biochem. Biophys Res. Commun*. **2017**, 20;487(1):167-172; Doi: 10.1016/j.bbrc.2017.03.171.

Wang, Z.; Ding, X.; Cao, F.; Zhang, X.; Wu, J. Bone Mesenchymal Stem Cells Promote Extracellular Matrix Remodeling of Degenerated Nucleus Pulposus Cells via the miR-101-3p/EIF4G2 Axis. *Front. Bioeng. Biotechnol.* **2021**, 27;9:642502; Doi: 10.3389/fbioe.2021.642502.

Wang, Z.; Zuo, Y.; Gao, Z. CircANKRD11 Knockdown Protects HPMECs from Cigarette Smoke Extract-Induced Injury by Regulating miR-145-5p/BRD4 Axis. *Int. J. Chron. Obstruct Pulmon Dis.* **2021**, 1;16:887-899; Doi: 10.2147/COPD.S300332.

Wei, B.; Liu, Y.S.; Guan, H.X. MicroRNA-145-5p attenuates high glucose-induced apoptosis by targeting the Notch signaling pathway in podocytes. *Exp. Ther. Med*. **2020**, 19(3):1915-1924; Doi: 10.3892/etm.2020.8427.

Wei, C.M.; Zhao, X.F.; Qiu, H.B.; Ming, Z.; Liu, K.; Yan, J. The long non-coding RNA PVT1/miR-145-5p/ITGB8 axis regulates cell proliferation, apoptosis, migration and invasion in non-small cell lung cancer cells. *Neoplasma*. **2020**, 67(4):802-812; Doi: 10.4149/neo_2020_190723N657.

Wei, Q.; Lv, F.; Zhang, H.; Wang, X.; Geng, Q.; Zhang, X.; et al. MicroRNA-101-3p inhibits fibroblast-like synoviocyte proliferation and inflammation in rheumatoid arthritis by targeting PTGS2. *Biosci. Rep*. **2020**, 31;40(1):BSR20191136; Doi: 10.1042/BSR20191136.

Wu, G.; Tan, J.; Li, J.; Sun, X.; Du, L.; Tao, S. miRNA-145-5p induces apoptosis after ischemia-reperfusion by targeting dual specificity phosphatase 6. *J. Cell Physiol*. **2019**; Doi: 10.1002/jcp.28291.

Wu, N.; Zhang, X.; Du, S.; Chen, D.; Che, R. Upregulation of miR-335 ameliorates myocardial ischemia reperfusion injury via targeting hypoxia inducible factor 1-alpha subunit inhibitor. *Am. J. Transl. Res.* **2018**, 15, 10(12):4082-4094.

Wu, X.; Zhou, J.; Wu, Z.; Chen, C.; Liu, J.; Wu, G.; et al. miR-101-3p Suppresses HOX Transcript Antisense RNA (HOTAIR)-Induced Proliferation and Invasion Through Directly Targeting SRF in Gastric Carcinoma Cells. *Oncol. Res.* **2017**, 21;25(8):1383-1390; Doi: 10.3727/096504017X14879366402279.

Xiaoping, L.; Zhibin, Y.; Wenjuan, L.; Zeyou, W.; Gang, X.; Zhaohui, L.; et al. CPEB1, a histone-modified hypomethylated gene, is regulated by miR-101 and involved in cell senescence in glioma. *Cell Death Dis*. **2013**, 20;4(6):e675; Doi: 10.1038/cddis.2013.197.

Xie, F.W.; Liu, J.C. LncRNA SNHG12 regulates the miR-101-3p/CUL4B axis to mediate the proliferation, migration and invasion of non-small cell lung cancer. *Kaohsiung J. Med. Sci*. **2021**, 37(8):664-674; Doi: 10.1002/kjm2.12389.

Xie, H.; Zhao, J.; Wan, J.; Zhao, J.; Wang, Q.; Yang, X.; et al. Long non-coding RNA AC245100.4 promotes prostate cancer tumorigenesis via the microRNA-145-5p/RBBP5 axis. *Oncol Rep*. **2021**, 45(2):619-629; Doi: 10.3892/or.2020.7894.

Xie, Y.; Deng, H.; Wei, R.; Sun, W.; Qi, Y.; Yao, S.; et al. Overexpression of miR-335 inhibits the migration and invasion of osteosarcoma by targeting SNIP1. *Int. J. Biol. Macromol*. **2019**, 15;133:137-147; Doi: 10.1016/j.ijbiomac.2019.04.016.

Xie, Y.; Wang, L., Yang, D. CircEPSTI1 Promotes the Progression of Non-Small Cell Lung Cancer Through miR-145/HMGB3 Axis. *Cancer Manag. Res*. **2020**, 4;12:6827-6836; Doi: 10.2147/CMAR.S252893.

Xin, Y.; Tang, L.; Chen, J.; Chen, D.; Wen, W.; Han, F. Inhibition of miR-101-3p protects against sepsis-induced myocardial injury by inhibiting MAPK and NF-kappaB pathway activation via the upregulation of DUSP1. *Int. J. Mol. Med*. **2021**, 47(3):20; Doi: 10.3892/ijmm.2021.4853.

Xiong, X.; Sun, D.; Chai, H.; Shan, W.; Yu, Y.; Pu, L.; Cheng, F. MiR-145 functions as a tumor suppressor targeting NUAK1 in human intrahepatic cholangiocarcinoma. *Biochem. Biophys Res. Commun*. **2015**, 18;465(2):262-9; Doi: 10.1016/j.bbrc.2015.08.013.

Xu, G.; Li, J.Y. Differential expression of PDGFRB and EGFR in microvascular proliferation in glioblastoma. *Tumour Biol.* **2016**, 37(8):10577-86; Doi: 10.1007/s13277-016-4968-3.

Xu, K.; Li, S.; Yang, Q.; Zhou, Z.; Fu, M.; Yang, X.; et al. MicroRNA-145-5p targeting of TRIM2 mediates the apoptosis of retinal ganglion cells via the PI3K/AKT signaling pathway in glaucoma. *J. Gene Med.* **2021**, 23(11):e3378; Doi: 10.1002/jgm.3378.

Xu, Q.; Liu, L.Z.; Qian, X.; Chen, Q.; Jiang, Y.; Li, D.; Lai, L.; Jiang, B.H. MiR-145 directly targets p70S6K1 in cancer cells to inhibit tumor growth and angiogenesis. *Nucleic Acids Res*. **2012**, 40(2):761-74; Doi: 10.1093/nar/gkr730.

Xu, X.; Liu, C.; Bao, J. Hypoxia-induced hsa-miR-101 promotes glycolysis by targeting TIGAR mRNA in clear cell renal cell carcinoma. *Mol. Med. Rep*. **2017**, 15(3):1373-1378; Doi: 10.3892/mmr.2017.6139.

Xu, Z.Y.; Peng, J.; Shi, Z.Z.; Chen, X.L.; Cheng, H.Z.; Wang, H.; et al. Silencing linc00662 inhibits cell proliferation and colony formation of lung cancer cells via regulating the miR-145-5p-PAFAH1B2 axis. *Biochem. Cell Biol*. **2021**, 99(3):330-338; Doi: 10.1139/bcb-2019-0396.

Xue, P.; Huang, S.; Han, X.; Zhang, C.; Yang, L.; Xiao, W.; et al. Exosomal miR-101-3p and miR-423-5p inhibit medulloblastoma tumorigenesis through targeting FOXP4 and EZH2. *Cell Death Differ*. **2022**, 29(1):82-95; Doi: 10.1038/s41418-021-00838-4.

Yang, A.; Liu, X.; Liu, P.; Feng, Y.; Liu, H.; Gao, S.; et al. LncRNA UCA1 promotes development of gastric cancer via the miR-145/MYO6axis. *Cell Mol. Biol. Lett*. **2021**, 8;26(1):33; Doi: 10.1186/s11658-021-00275-8.

Yang, D.; Lutter, D.; Burtscher, I.; Uetzmann, L.; Theis, F.J.; Lickert, H. miR-335 promotes mesendodermal lineage segregation and shapes a transcription factor gradient in the endoderm. *Development* **2014**, 141(3):514-25; Doi: 10.1242/dev.104232.

Yang, Q.; Yu, W.; Han, X. Overexpression of microRNA-101 causes anti-tumor effects by targeting CREB1 in colon cancer. *Mol. Med. Rep*. **2019**, 19(4):3159-3167; Doi: 10.3892/mmr.2019.9952.

Yang, W.; Yin, R.; Zhu, X.; Yang, S.; Wang, J.; Zhou, Z.; et al. Mesenchymal stem-cell-derived exosomal miR-145 inhibits atherosclerosis by targeting JAM-A. *Mol. Ther. Nucleic Acids*. **2020**, 4;23:119-131; Doi: 10.1016/j.omtn.2020.10.037.

Yang, X.W.; Zhang, L.J.; Huang, X.H.; Chen, L.Z.; Su, Q.; Zeng, W.T.; Li, W.; Wang, Q. miR-145 suppresses cell invasion in hepatocellular carcinoma cells: miR-145 targets ADAM17. *Hepatol. Res*. **2014**, 44(5):551-559; Doi: 10.1111/hepr.12152.

Yang, X.Y.; Sheng, Y. miR-101 Represses T-Cell Acute Lymphoblastic Leukemia by Targeting CXCR7/STAT3 Axis. *Oncol. Res.* **2019**, 23;27(9):997-1006; Doi: 10.3727/096504018X15439207752093.

Yang, Y.; Li, X.J.; Li, P., Guo, X.T. MicroRNA-145 regulates the proliferation, migration and invasion of human primary colon adenocarcinoma cells by targeting MAPK1. *Int. J. Mol. Med*. **2018**, 42(6):3171-3180; Doi: 10.3892/ijmm.2018.3904.

Yang, Y.; Wang, F.; Huang, H.; Zhang, Y.; Xie, H.; Men, T. lncRNA SLCO4A1-AS1 promotes growth and invasion of bladder cancer through sponging miR-335-5p to upregulate OCT4. *Onco. Targets Ther*. **2019**, 18;12:1351-1358; Doi: 10.2147/OTT.S191740.

Yao, L.; Li, M.; Hu, J.; Wang, W.; Gao, M. MiRNA-335-5p negatively regulates granulosa cell proliferation via SGK3 in PCOS. *Reproduction.* **2018**, 156(5):439-449; Doi: 10.1530/REP-18-0229.

Yao, Y.; Wang, Y.; Li, L.; Xiang, X.; Li, J.; Chen, J.; et al. Down-regulation of interferon regulatory factor 2 binding protein 2 suppresses gastric cancer progression by negatively regulating connective tissue growth factor. *J. Cell Mol. Med.* **2019**, 23(12):8076-8089; Doi: 10.1111/jcmm.14677.

Ye, Y.; Hao, J.; Hong, Z.; Wu, T.; Ge, X.; Qian, B.; et al. Downregulation of MicroRNA-145-5p in Activated Microglial Exosomes Promotes Astrocyte Proliferation by Removal of Smad3 Inhibition. *Neurochem Res*. **2022**, 47(2):382-393; Doi: 10.1007/s11064-021-03446-3.

Ye, Y.; Li, Z.; Feng, Q.; Chen, Z.; Wu, Z.; Wang, J.; et al. Downregulation of microRNA-145 may contribute to liver fibrosis in biliary atresia by targeting ADD3. *PLoS One*. **2017**, 13;12(9):e0180896; Doi: 10.1371/journal.pone.0180896.

Ye, Z.; Yin, S.; Su, Z.; Bai, M.; Zhang, H.; Hei, Z.; Cai, S. Downregulation of miR-101 contributes to epithelial-mesenchymal transition in cisplatin resistance of NSCLC cells by targeting ROCK2. *Oncotarget*. **2016**, 7(25):37524-37535; Doi: 10.18632/oncotarget.6852.

Yu, C.; Li, B.; Wang, J.; Zhang, Z.; Li, S.; Lei, S.; Wang, Q. miR-145-5p Modulates Gefitinib Resistance by Targeting NRAS and MEST in Non-Small Cell Lung Cancer. *Ann. Clin. Lab. Sci*. **2021**, 51(5):625-637.

Yu, C.; Ying, J.; Yu, K.; Shen, W.; Jiang, M. Circ_0074027 Contributes to Nonsmall Cell Lung Cancer Progression by Upregulating CUL4B Expression Through miR-335-5p. *Cancer Biother. Radiopharm*. **2020**; Doi: 10.1089/cbr.2020.3579.

Yu, W.; Ding, J.; He, M.; Chen, Y.; Wang, R.; Han, Z.; et al. Estrogen receptor beta promotes the vasculogenic mimicry (VM) and cell invasion via altering the lncRNA-MALAT1/miR-145-5p/NEDD9 signals in lung cancer. *Oncogene.* **2019**, 38(8):1225-1238; Doi: 10.1038/s41388-018-0463-1.

Yuan, M.; Zhang, L.; You, F.; Zhou, J.; Ma, Y.; Yang, F.; Tao, L. MiR-145-5p regulates hypoxia-induced inflammatory response and apoptosis in cardiomyocytes by targeting CD40. *Mol. Cell Biochem*. **2017**, 431(1-2):123-131; Doi: 10.1007/s11010-017-2982-4.

Yuan, X.; Sun, Z.; Cui, C. Knockdown of lncRNA HOTTIP Inhibits Retinoblastoma Progression by Modulating the miR-101-3p/STC1 Axis. *Technol. Cancer Res. Treat*. **2021**, 20:1533033821997831; Doi: 10.1177/1533033821997831.

Yuan, Y.; Xue, C.; Wu, Q.; Wang, M.; Liu, J.; Zhang, L.; et al. The miR-145-5p/CD36 pathway mediates PCB2-induced apoptosis in MCF-7 cells. *Genes Genomics*. **2021**, 43(2):161-171; Doi: 10.1007/s13258-020-01025-4.

Zeng, H.; Huang, Y.; Liu, Q.; Liu, H.; Long, T.; Zhu, C.; Wu, X. MiR-145 suppresses the motility of prostate cancer cells by targeting cadherin-2. *Mol. Cell Biochem*. **2021**, 476(10):3635-3646; Doi: 10.1007/s11010-021-04188-0.

Zeng, H.; Li, L.; Gao, Y.; Wu, G.; Hou, Z.; Liu, S. Long noncoding RNA UCA1 regulates HCV replication and antiviral response via miR-145-5p/SOCS7/IFN pathway. *Int. J. Biol. Sci.* **2021**, 5;17(11):2826-2840; Doi: 10.7150/ijbs.59227.

Zhan, M.; Zhao, X.; Wang, H.; Chen, W.; Xu, S.; Wang, W.; Shen, H.; Huang, S.; Wang, J. miR-145 sensitizes gallbladder cancer to cisplatin by regulating multidrug resistance associated protein 1. *Tumour Biol.* **2016**, 37(8):10553-62; Doi: 10.1007/s13277-016-4957-6.

Zhang, D.; Yang, N. MiR-335-5p Inhibits Cell Proliferation, Migration and Invasion in Colorectal Cancer through Downregulating LDHB. *J. BUON.* **2019**, 24(3):1128-1136.

Zhang, H.; Jiang, M.; Liu, Q.; Han, Z.; Zhao, Y.; Ji, S. miR-145-5p inhibits the proliferation and migration of bladder cancer cells by targeting TAGLN2. *Oncol Lett*. **2018**, 16(5):6355-6360; Doi: 10.3892/ol.2018.9436.

Zhang, H.; Wang, X.; Hu, B.; Zhang, F.; Wei, H.; Li, L. Circular RNA ZFR accelerates non-small cell lung cancer progression by acting as a miR-101-3p sponge to enhance CUL4B expression. *Artif. Cells Nanomed. Biotechnol.* **2019**, 47(1):3410-3416; Doi: 10.1080/21691401.2019.1652623.

Zhang, J.; Cui, C.; Xu, H. Downregulation of miR-145-5p elevates retinal ganglion cell survival to delay diabetic retinopathy progress by targeting FGF5. *Biosci Biotechnol Biochem*. **2019**, 83(9):1655-1662; Doi: 10.1080/09168451.2019.1630251.

Zhang, J.; Huang, J.; Wang, X.; Chen, W.; Tang, Q.; Fang, M.; Qian, Y. CMIP is oncogenic in human gastric cancer cells. *Mol. Med. Rep.* **2017**, 16(5):7277-7286; Doi: 10.3892/mmr.2017.7541.

Zhang, J.; Li, Z.; Huang, J.; Chen, S.; Yin, H.; Tian, J.; Qu, L. miR-101 inhibits feline herpesvirus 1 replication by targeting cellular suppressor of cytokine signaling 5 (SOCS5). *Vet. Microbiol.* **2020**, 245:108707; Doi: 10.1016/j.vetmic.2020.108707.

Zhang, J.; Liu, J.; Liu, Y.; Wu, W.; Li, X.; Wu, Y.; et al. miR-101 represses lung cancer by inhibiting interaction of fibroblasts and cancer cells by down-regulating CXCL12. *Biomed. Pharmacother*. **2015**, 74:215-21; Doi: 10.1016/j.biopha.2015.08.013.

Zhang, L.; Wang, X.; Li, Y.; Han, J.; Gao, X.; Li, S.; et al. c-Myb facilitates immune escape of esophageal adenocarcinoma cells through the miR-145-5p/SPOP/PD-L1 axis. *Clin. Transl. Med.* **2021**, 11(9):e464; Doi: 10.1002/ctm2.464.

Zhang, L.L.; Zhang, L.F.; Guo, X.H.; Zhang, D.Z.; Yang, F.; Fan, Y.Y. Downregulation of miR-335-5p by Long Noncoding RNA ZEB1-AS1 in Gastric Cancer Promotes Tumor Proliferation and Invasion. *DNA Cell Biol.* **2018**, 37(1):46-52; Doi: 10.1089/dna.2017.3926.

Zhang, M.; Liu, W.; Zhang, Q.; Hu, H. miR-101-3p Contributes to alpha-Synuclein Aggregation in Neural Cells through the miR-101-3p/SKP1/PLK2 Pathway. *J. Healthc. Eng.* **2021**, 12;2021:6147434; Doi: 10.1155/2021/6147434.

Zhang, P.; Yang, X.; Wang, L.; Zhang, D.; Luo, Q.; Wang, B. Overexpressing miR-335 inhibits DU145 cell proliferation by targeting early growth response 3 in prostate cancer. *Int. J. Oncol.* **2019**, 54(6):1981-1994; Doi: 10.3892/ijo.2019.4778.

Zhang, S.; Wu, J.; Zhu, X.; Song, H.; Ren, L.; Tang, Q.; et al. A novel approach to identify the mechanism of miR-145-5p toxicity to podocytes based on the essential genes targeting analysis. *Mol. Ther. Nucleic Acids.* **2021**, 20;26:749-759; Doi: 10.1016/j.omtn.2021.09.005.

Zhang, X.; Gao, D.; Fang, K.; Guo, Z.; Li, L. Med19 is targeted by miR-101-3p/miR-422a and promotes breast cancer progression by regulating the EGFR/MEK/ERK signaling pathway. *Cancer Lett.* **2019**, 1;444:105-115; Doi: 10.1016/j.canlet.2018.12.008.

Zhao, H.; Tang, H.; Huang, Q.; Qiu, B.; Liu, X.; Fan, D.; et al. MiR-101 targets USP22 to inhibit the tumorigenesis of papillary thyroid carcinoma. *Am. J. Cancer Res.* **2016**, 1;6(11):2575-2586.

Zhao, J.; Su, L.; Jiang, J. Long Non-Coding RNA Paternally Expressed Imprinted Gene 10 (PEG10) Elevates Diffuse Large B-Cell Lymphoma Progression by Regulating Kinesin Family Member 2A (KIF2A) via Targeting MiR-101-3p. *Med. Sci. Monit.* **2020**, 25;26:e922810; Doi: 10.12659/MSM.922810.

Zhao, W.; Zhao, Y.; Chen, L.; Sun, Y.; Fan, S. miR-335-5p Inhibits Progression of Uterine Leiomyoma by Targeting ARGLU1. *Comput. Math Methods Med.* **2022**, 17;2022:2329576; Doi: 10.1155/2022/2329576.

Zhao, X.; Li, S.; Wang, Z.; Bai, N.; Feng, Y. miR-101-3p negatively regulates inflammation in systemic lupus erythematosus via MAPK1 targeting and inhibition of the NF-kappaB pathway. *Mol. Med. Rep.* **2021**, 23(5):359; Doi: 10.3892/mmr.2021.11998.

Zhao, Y.; Ai, Y. Knockdown of lncRNA MALAT1 alleviates bupivacaine-induced neurotoxicity via the miR-101-3p/PDCD4 axis. *Life Sci.* **2019**, 1;232:116606; Doi: 10.1016/j.lfs.2019.116606.

Zhao, Y.; Yu, Z.; Ma, R.; Zhang, Y.; Zhao, L.; Yan, Y.; et al. lncRNA-Xist/miR-101-3p/KLF6/C/EBPalpha axis promotes TAM polarization to regulate cancer cell proliferation and migration. *Mol. Ther. Nucleic Acids.* **2020**, 10;23:536-551; Doi: 10.1016/j.omtn.2020.12.005.

Zheng, F.; Xu, R. CircPVT1 contributes to chemotherapy resistance of lung adenocarcinoma through miR-145-5p/ABCC1 axis. *Biomed. Pharmacother.* **2020**, 124:109828; Doi: 10.1016/j.biopha.2020.109828.

Zheng, S.Q.; Li, Y.X.; Zhang, Y.; Li, X.; Tang, H. MiR-101 regulates HSV-1 replication by targeting ATP5B. *Antiviral Res.* **2011**, 89(3):219-26; Doi: 10.1016/j.antiviral.2011.01.008.

Zheng, Z.; Chen, Z.; Zhong, Q.; Zhu, D.; Xie, Y.; Shangguan, W.; Xie, W. CircPVT1 promotes progression in clear cell renal cell carcinoma by sponging miR-145-5p and regulating TBX15 expression. *Cancer Sci.* **2021**, 112(4):1443-1456; Doi: 10.1111/cas.14814.

Zhong, X.; Wen, X.; Chen, X.; Gu, N.; Yu, X.; Sui, K. Long non-coding RNA KCNQ1OT1 promotes the progression of gastric cancer via the miR-145-5p/ARF6 axis. *J. Gene Med*. **2021**, 23(5):e3330; Doi: 10.1002/jgm.3330.

Zhou, H.; Li, L.; Wang, Y.; Wang, D. Long non-coding RNA SNHG6 promotes tumorigenesis in melanoma cells via the microRNA-101-3p/RAP2B axis. *Oncol. Lett.* **2020**, 20(6):323; Doi: 10.3892/ol.2020.12186.

Zhou, J.; Zhang, X.; Li, W.; Chen, Y.J. MicroRNA-145-5p regulates the proliferation of epithelial ovarian cancer cells via targeting SMAD4. *Ovarian Res*. **2020**, 4;13(1):54; Doi: 10.1186/s13048-020-00656-1.

Zhou, K.; Song, B.; Wei, M.; Fang, J.; Xu, Y. MiR-145-5p suppresses the proliferation, migration and invasion of gastric cancer epithelial cells via the ANGPT2/NOD_LIKE_RECEPTOR axis. *Cancer Cell Int*. **2020**, 28;20:416; Doi: 10.1186/s12935-020-01483-6.

Zhou, T.; Chen, S.; Mao, X. miR-145-5p affects the differentiation of gastric cancer by targeting KLF5 directly. *J. Cell Physiol*. **2019**, 234(5):7634-7644; Doi: 10.1002/jcp.27525.

Zhou, W.; Ji, L.; Liu, X.; Tu, D.; Shi, N.; Yangqu, W.; et al. AIFM1, negatively regulated by miR-145-5p, aggravates hypoxia-induced cardiomyocyte injury. *Biomed. J.* **2021**, 1:S2319-4170(21)00172-4; Doi: 10.1016/j.bj.2021.11.012.

Zhou, X.M.; Sun, R.; Luo, D.H.; Sun, J.; Zhang, M.Y.; Wang, M.H.; et al. Upregulated TRIM29 promotes proliferation and metastasis of nasopharyngeal carcinoma via PTEN/AKT/mTOR signal pathway. *Oncotarget.* **2016**, 22;7(12):13634-50; Doi: 10.18632/oncotarget.7215.

Zhou, Z.; Xu, H.; Duan, Y.; Liu, B. MicroRNA-101 suppresses colorectal cancer progression by negative regulation of Rap1b. *Oncol. Lett.* **2020**, 20(3):2225-2231; Doi: 10.3892/ol.2020.11791.

Zhu, J.; Li, Z. Overexpression of miR-101 promotes TRAIL-induced mitochondrial apoptosis in papillary thyroid carcinoma by targeting c-met and MCL-1. *Oncotarget*. **2017**, 23;8(65):108665-108675; Doi: 10.18632/oncotarget.21215.

Zhu, J.; Zhang, F. Circular RNA VANGL1 knockdown suppressed viability, promoted apoptosis, and increased doxorubicin sensitivity through targeting miR-145-5p to regulate SOX4 in bladder cancer cells. *Open Med.* **2021**, 6;16(1):1010-1021; Doi: 10.1515/med-2021-0299.

Zhu, L.; Chen, Y.; Nie, K.; Xiao, Y.; Yu, H. MiR-101 inhibits cell proliferation and invasion of pancreatic cancer through targeting STMN1. *Cancer Biomark*. **2018**, 23(2):301-309; Doi: 10.3233/CBM-181675.

Zhu, W.; Li, Z.; Xiong, L.; Yu, X.; Chen, X.; Lin, Q. FKBP3 Promotes Proliferation of Non-Small Cell Lung Cancer Cells through Regulating Sp1/HDAC2/p27. *Theranostics* **2017**, 22;7(12):3078-3089; Doi: 10.7150/thno.18067.

Zhu, X.; Li, Y.; Xie, C.; Yin, X.; Liu, Y.; Cao, Y.; et al. miR-145 sensitizes ovarian cancer cells to paclitaxel by targeting Sp1 and Cdk6. *Int. J. Cancer.* **2014**, 15;135(6):1286-96; Doi: 10.1002/ijc.28774.

Zhu, Z.; Wu, Q.; Zhang, M.; Tong, J.; Zhong, B.; Yuan, K. Hsa_circ_0016760 exacerbates the malignant development of non-small cell lung cancer by sponging miR-145-5p/FGF5. *Oncol. Rep*. **2021**, 45(2):501-512; Doi: 10.3892/or.2020.7899.

Zhuang, S.; Liu, F.; Wu, P. Upregulation of long noncoding RNA TUG1 contributes to the development of laryngocarcinoma by targeting miR-145-5p/ROCK1 axis. *J. Cell Biochem*. **2019**, 120(8):13392-13402; Doi: 10.1002/jcb.28614.

Zhuang, W.; Niu, T.; Li, Z. MicroRNA miR-145-5p regulates cell proliferation and cell migration in colon cancer by inhibiting chemokine (C-X-C motif) ligand 1 and integrin alpha2. *Bioengineered*. **2021**, 12(2):9909-9917; Doi: 10.1080/21655979.2021.2000243.

Zhuang, X.; Chen, B.; Huang, S.; Han, J.; Zhou, G.; Xu, S.; et al. Hypermethylation of miR-145 promoter-mediated SOX9-CLDN8 pathway regulates intestinal mucosal barrier in Crohn's disease. *BioMedicine* **2022**, 76:103846; Doi: 10.1016/j.ebiom.2022.103846.

Zou, C.; Xu, Q.; Mao, F.; Li, D.; Bian, C.; Liu, L.Z.; et al. MiR-145 inhibits tumor angiogenesis and growth by N-RAS and VEGF. *Cell Cycle*. **2012**, 1;11(11):2137-45; Doi: 10.4161/cc.20598.

Zou, J.; Wu, K.; Lin, C.; Jie, Z.G. LINC00319 acts as a microRNA-335-5p sponge to accelerate tumor growth and metastasis in gastric cancer by upregulating ADCY3. *Am. J. Physiol. Gastrointest Liver Physiol*. **2020**, 1;318(1):G10-G22; Doi: 10.1152/ajpgi.00405.2018.

Zu, Y.; Ban, J.; Xia, Z.; Wang, J.; Cai, Y.; Ping, W.; Sun, W. Genetic variation in a miR-335 binding site in BIRC5 alters susceptibility to lung cancer in Chinese Han populations. *Biochem. Biophys Res. Commun*. **2013**, 11;430(2):529-34; Doi: 10.1016/j.bbrc.2012.12.001.

TABLE S3

Levels of CFTR protein content in Calu-3 cells treated with PNA-a145, PNA-a101, and PNA-a335 administrated singularly or in combination

Treatment	CFTR (Fold average)	Standard Deviation (SD)
PNA-a145	1.94	0.19
PNA-a101	1.96	0.07
PNA-a335	1.57	0.14
PNA-a145 + PNA-a101	9.33	0.99
PNA-a145 + PNA-a335	4.95	0.26
PNA-a101 + PNA-a335	1.93	0.20
